# *Saccharomyces cerevisiae FLO1* Gene Demonstrates Genetic Linkage to Increased Fermentation Rate at Low Temperatures

**DOI:** 10.1534/g3.116.037630

**Published:** 2017-01-30

**Authors:** Rebecca C. Deed, Bruno Fedrizzi, Richard C. Gardner

**Affiliations:** * School of Chemical Sciences, University of Auckland, 1010, New Zealand; † School of Biological Sciences, University of Auckland, 1010, New Zealand

**Keywords:** fermentation kinetics, genetic linkage analysis, low temperature, wine

## Abstract

Low fermentation temperatures are of importance to food and beverage industries working with *Saccharomyces cerevisiae*. Therefore, the identification of genes demonstrating a positive impact on fermentation kinetics is of significant interest. A set of 121 mapped F_1_ progeny, derived from a cross between haploid strains BY4716 (a derivative of the laboratory yeast S288C) and wine yeast RM11-1a, were fermented in New Zealand Sauvignon Blanc grape juice at 12.5°. Analyses of five key fermentation kinetic parameters among the F_1_ progeny identified a quantitative trait locus (QTL) on chromosome I with a significant degree of linkage to maximal fermentation rate (*V*_max_) at low temperature. Independent deletions of two candidate genes within the region, *FLO1* and *SWH1*, were constructed in the parental strains (with S288C representing BY4716). Fermentation of wild-type and deletion strains at 12.5 and 25° confirmed that the genetic linkage to *V*_max_ corresponds to the S288C version of the *FLO1* allele, as the absence of this allele reduced *V*_max_ by ∼50% at 12.5°, but not at 25°. Reciprocal hemizygosity analysis (RHA) between S288C and RM11-1a *FLO1* alleles did not confirm the prediction that the S288C version of *FLO1* was promoting more rapid fermentation in the opposing strain background, suggesting that the positive effect on *V*_max_ derived from S288C *FLO1* may only provide an advantage in haploids, or is dependent on strain-specific *cis* or *trans* effects. This research adds to the growing body of evidence demonstrating the role of *FLO1* in providing stress tolerance to *S. cerevisiae* during fermentation.

The use of low temperatures (<18°) for many commercially important fermentative processes carried out by *Saccharomyces cerevisiae*, including baking, white winemaking, and rosé winemaking, is currently the industry norm. Although there is a widely held belief by winemakers and oenologists that low fermentation temperatures increase white wine quality (Uchimoto and Cruess 1952; Killian and Ough 1979; Llauradó *et al.* 2002; Molina *et al.* 2007), there is also an increase in the risk of stuck and sluggish fermentations, longer lag phase, and a decrease in the rate of yeast growth and fermentation, slowing down industrial processes and increasing financial costs (Charoenchai *et al.* 1998; Llauradó *et al.* 2002; Torija *et al.* 2003; Coleman *et al.* 2007; Chiva *et al.* 2012). Therefore, the identification of genes encoding proteins with the ability to confer cold tolerance during fermentation can be useful for selecting *S. cerevisiae* strains to be used in industries working with low fermentation temperatures, resulting in improved efficiencies and lower costs.

Low temperature environments are highly stressful for yeast, impacting on a multitude of cellular and metabolic processes: a reduction in membrane fluidity; a reduction in oxygen solubility; changes in nutrient uptake, transport and consumption; an increase in the biosynthesis of protective compounds; and a reduction in the rate of biochemical reactions (Sahara *et al.* 2002; Schade *et al.* 2004; Aguilera *et al.* 2007; Tai *et al.* 2007; Pizarro *et al.* 2008; Chiva *et al.* 2012). Environments that promote fermentation already contain many stresses that impact on yeast cells, *e.g.*, high sugar, ethanol and toxic fatty acid concentrations, low pH, reduced concentrations of oxygen, and limited nitrogen. Therefore, the added stress of low fermentation temperatures requires an even greater response by *S. cerevisiae*, corresponding to altered transcription of ∼500–1000 genes depending on the strain and conditions used (Beltran *et al.* 2006; Deed *et al.* 2015). The transcriptional response to low temperature fermentation is initiated in two steps, first via the induction of cold-specific stress genes, followed by the more generalized environmental stress response and fermentation stress response (Gasch and Werner-Washburne 2002; Beltran *et al.* 2006; Marks *et al.* 2008; Deed *et al.* 2015). It has been well documented that different *S. cerevisiae* strains vary greatly in their ability to grow and ferment at lower temperatures (Charoenchai *et al.* 1998; Torija *et al.* 2003), and it has been suggested that these phenotypic differences are due to strain differences in gene expression, particularly via variation in gene promoter regions and the expression of transcription factors (Beltran *et al.* 2006; Chiva *et al.* 2012; Treu *et al.* 2014; Deed *et al.* 2015).

We have carried out genetic linkage analysis, using a set of 121 completely mapped (>99% of the genome) F_1_ progeny from a cross between haploid strains BY4716 and RM11-1a [denoted as BY and RM respectively in Brem *et al.* (2002)], to identify QTL with a positive influence on yeast fermentation kinetics at low temperature (12.5°). A region on chromosome I showed statistically significant genetic linkage to *V*_max_ among the F_1_ progeny, and gene deletions and RHA were used to investigate the causative gene within this region.

## Materials and Methods

### S. cerevisiae strains

We utilized 121 segregant F_1_ progeny derived from a cross between laboratory strain BY4716 (*MAT*α, *lys2*-Δ*0*), an isogenic derivative of laboratory strain S288C (Brachmann *et al.* 1998), and RM11-1a (*MATa*, *leu2*-Δ*0*, *ura3*-Δ*0*, *HO*::*KanMX*), a haploid derived from the wild vineyard-associated isolate Bb32 (Mortimer *et al.* 1994). BY4716 × RM11-1a F_1_ progeny were generated by Brem *et al.* (2002) for linkage analysis using 2957 mapped loci (kindly gifted by E. Smith and L. Kruglyak, Princeton University). S288C (*MAT*α), representing the BY4716 parent, and the RM11-1a parent were used as reference strains to compare against fermentation phenotypes observed across the F_1_ progeny. Gene deletions and RHA were carried out in the S288C and RM11-1a strain backgrounds.

### Growth and fermentation conditions

BY4716 × RM11-1a F_1_ progeny, parental strains, and diploid F_1_ hybrids generated for RHA were fermented at 12.5° (and 25° for the RHA strains) in Sauvignon Blanc grape juice, containing ∼22° Brix and 281 mg L^−1^ yeast assimilable nitrogen (Pernod Ricard, Marlborough, New Zealand). Grape juice was sterilized via overnight incubation at 25° with 200 μl L^−1^ dimethyl dicarbonate and supplemented with the following amino acids: 10 × leucine (300 mg L^−1^), 10 × lysine (300 mg L^−1^), and 10 × uracil (100 mg L^−1^). Yeast cultures were propagated in yeast-peptone-dextrose medium (YPD) and incubated overnight at 28°, with orbital shaking at 150 rpm. Grape juice was mixed well before being used to make 8 ml aliquots in 13-ml ventilation cap polypropylene tubes to ensure an even distribution of grape solids. Fermentations were inoculated with 1 × 10^6^ cells ml^−1^ and a <0.5 mm^2^ pin-hole was punctured into each tube lid to allow for CO_2_ escape. Fermentations were monitored daily by measuring cumulative weight loss (g) (Bely *et al.* 1990). To reduce variability within triplicate fermentations, outliers were removed if they deviated from other replicates by >10% weight loss at three consecutive time points, after >50% total weight loss. Fermentations of RHA strains were performed in nonaplicates (*n* = 9).

### Analysis of fermentation kinetic parameters

Phenotypes for five fermentation-related kinetic variables, maximal fermentation rate (*V*_max_) (*dCO*_2_*/dt*), maximal acceleration rate (*A*_max_) (*d*^2^*CO*_2_*/dt*^2^), length of lag phase (h), final weight loss (g), and finishing time of alcoholic fermentation (*AF time*) (h), were determined from cumulative weight loss data, as per Marullo *et al.* (2006).

### Linkage analysis

Quantitative phenotypic data for the five fermentation kinetic parameters were sent to J. Bloom and J. Gerke (Princeton University) for QTL mapping and identification of relevant loci. Logarithm (base 10) of odds (LOD) scores were generated for 2957 genetic markers across the 16 *S. cerevisiae* chromosomes using R/QTL’s scanone function, and a nonparametric model to compare the likelihood of obtaining the phenotypic data if mapped loci are linked against the likelihood of obtaining the data by chance (Broman *et al.* 2003). GBrowse maps of chromosomal regions with LOD scores > 3 (significant with a 5% chance of error) were obtained from the *Saccharomyces* Genome Database (SGD) (http://www.yeastgenome.org/) to determine candidate open reading frames (ORFs) linked to *V*_max_ and lag phase.

### Gene deletions and RHA

Deletion of candidate genes, *FLO1* and *SWH1*, within the chromosome I QTL linked to *V*_max_, were constructed in S288C and RM11-1a using a modification of the Schiestl and Gietz (1989) lithium acetate yeast transformation protocol. The *KanMX* construct within the *HO* gene of RM11-1a was replaced with a hygromycin resistance (HGM^R^) cassette, *HphMX*, to allow for subsequent integration of *KanMX* into the two candidate genes. Transformation of haploid S288C and RM11-1a was performed independently to generate mutants with *KanMX* insertions in *FLO1* and *SWH1* using constructs amplified from the BY4743 EUROSCARF strains, *FLO1*Δ*YAR050W*::*KanMX* and *SWH1*Δ*YAR042W*::*KanMX*. Deletions of *FLO1* and *SWH1* were confirmed by PCR (list of oligonucleotide primers in Table 1). Crosses were made between wild-type S288C, RM11-1a, and *flo1* and *swh1* deletion mutants in order to construct diploid F_1_ hybrids for RHA (Steinmetz *et al.* 2002) (crosses shown in Table 2). A multiplex PCR to amplify 10 variable microsatellite markers and two mating type loci, *MATa* and *MAT*α, was used to ensure that the F_1_ hybrids were constructed correctly (Table 3) (Richards *et al.* 2009).

**Table 1:** Oligonucleotide primers used for gene deletions and RHA

Primer Name	Sequence (5′–3′)	Purpose
3′kanI-F	GGTCGCTATACTGCTGTC	Confirm integration of *KanMX* constructs
*HO*F2	TGCAGAAGCTTGTTGAAGCA	Amplify *HphMX* insertion within *HO*
*HO*R2	GCCGGTAACGCTTTTTGTAT	Amplify *HphMX* insertion within *HO*
*MATa*	ACTCCACTTCAAGTAAGAGTTTG	Amplify the *MATa* locus
*Mat*α	GCACGGAATATGGGACTACTTCG	Amplify the *MAT*α locus
*MatR*	AGTCACATCAAGATCGTTTATGG	Amplify the *MATa/*α locus
*FLO1*intL-F	CGGCACAGTTGAAAGAGTCA	Amplify *KanMX* from BY4743 *flo1* deletion strain with flanking regions of homology
*FLO1*intR-R	GGCGATGGTTCATTAATTGC	Amplify *KanMX* from BY4743 *flo1* deletion strain with flanking regions of homology
*FLO1*testL-F	GCCCTCACAAGAATTTGGAA	Flanking test primer used to confirm integration of *KanMX* into the *FLO1* locus of transformants
*FLO1*testR-R	TTCCTGGGAACGAAAAGCTA	Flanking test primer used to confirm integration of *KanMX* into the *FLO1* locus of transformants
*SWH1*intL-F	GCGTGTCCGGTTGAGTTTAT	Amplify *KanMX* from BY4743 *swh1* deletion strain with flanking regions of homology
*SWH1*intR2-R	TTGCAGCAATTCGTTCAAAG	Amplify *KanMX* from BY4743 *swh1* deletion strain with flanking regions of homology
*SWH1*testL2-F	GCCAGGACCGTCACTTGTAT	Flanking test primer used to confirm integration of *KanMX* into the *SWH1* locus of transformants

**Table 2:** Strains used to make crosses for RHA between S288C and RM11-1a for the *FLO1* and *SWH1* loci

Cross	Parent #1	Parent #2	F_1_ Hybrid Selection
R-*FS* × S-*FS*	RM11-1a (*HO*::*HphMX*; *MATa*)	**S288C (*MAT*α)**	*HGM^R^
R-*FS* × S-*fS*	RM11-1a (*HO*::*HphMX*; *MATa*)	S288C (*FLO1*::*KanMX*; *MAT*α)	HGM^R^; Kan^R^
R-*FS* × S-*Fs*	RM11-1a (*HO*::*HphMX*; *MATa*)	S288C (*SWH1*::*KanMX*; *MAT*α)	HGM^R^; Kan^R^
R-*fS* × S-*FS*	RM11-1a (*HO*::*HphMX*; *FLO1*::*KanMX*; *MATa*)	**S288C (*MAT*α)**	*HGM^R^; Kan^R^
R-*Fs* × S-*FS*	RM11-1a (*HO*::*HphMX*; *SWH1*::*KanMX*; *MATa*)	**S288C (*MAT*α)**	*HGM^R^; Kan^R^

The S288C parent strain in bold were added in 100 × excess of the RM11-1a parent, as S288C did not have any selectable markers that differed from RM11-1a. The F_1_ hybrid selections marked with * could result in the presence of the RM11-1a parent, as well as the F_1_ hybrid. The R-*FS* × S-*FS* cross was included as a control. HGM^R^, hygromycin resistance; Kan^R^, kanamycin resistance.

**Table 3:** Microsatellite confirmation of F_1_ hybrid strains between S288C and RM11-1a for RHA

Strain	*C3*	*C5*	*C8*	*C4*	*091c*	*AT4*	*AT2*	*Scaat3*	*009c*	*267c*	α	*a*
S288C	120	174	130	240	302	296	357	407	443	415	468	—
RM11-1a	121	139	146	259	260	296	364	381	419	427	—	492
R-*Fs* × S-*FS*	120	139	130	240	260	296	358	381	419	415	468	492
	121	174	146	259	303	296	364	407	443	427		
R-*fS* × S-*FS*	120	139	130	240	260	296	358	381	419	415	468	492
	121	174	146	259	303	296	364	407	443	427		
R-*FS* × S*Fs*	120	138	130	240	260	296	358	381	419	415	468	492
	121	174	146	259	303	296	363	413	443	427		
R-*FS* × S*fS*	120	138	130	240	260	296	358	381	419	415	468	492
	121	174	146	259	302	296	363	407	443	427		
R-*FS* × S-*FS*	120	139	130	240	260	296	358	381	419	415	468	492
	121	174	146	259	302	296	363	407	443	427		

Numbers are band sizes in base pairs. The 12 loci detected correspond to 10 variable microsatellite loci and two mating type loci, *MATa* and *MAT*α, as described in Richards *et al.* (2009).

### Data availability

All strains are available upon request. Supplemental Material: Table S1 contains the values for the kinetic parameters for all individuals. Table S2 contains the list of ORFs identified either side of each LOD >3 peak marker. File S1 contains the LOD scores for all individuals across the five fermentation parameters. File S2 contains Clustal alignments. 

## Results

### Fermentation of 121 mapped F_1_ progeny identified genes linked to fermentation rate and lag phase

**Figure 1:**
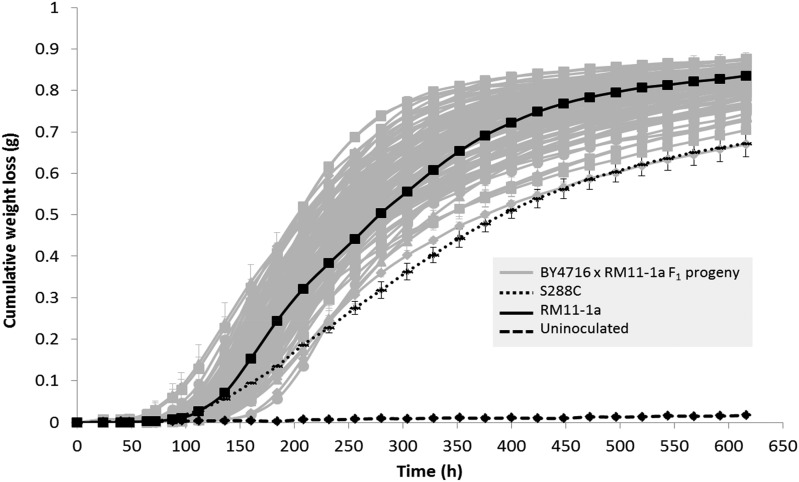
Average cumulative weight loss (g) of 121 BY4716 × RM11-1a F_1_ progeny and parental reference strains S288C and RM11-1a. Strains were fermented in Sauvignon Blanc juice at 12.5°. BY4716 × RM11-1a F_1_ progeny = gray. S288C = black, small dashed line. RM11-1a = black solid line. Uninoculated = black dashed line, *n* = 3, error bars represent 95% C.I.s.

Cumulative weight loss was measured for 616 hr throughout the fermentation of 121 BY4716 × RM11-1a F_1_ progeny at 12.5° (Figure 1). As expected, the RM11-1a parental strain demonstrated superior fermentation performance compared to the S288C parental reference. F_1_ progeny demonstrated sufficient phenotypic variation for genetic mapping with fermentation curves covering the full range between both parents. Positive heterosis was also evident, with some F_1_ progeny exhibiting improved fermentation performance compared to RM11-1a. After removal of outliers (triplicate fermentations that deviated by >10% weight loss), 6/121 of the F_1_ progeny were analyzed only in duplicate (3D, 5B, 6A, 8G, 10G, and 3A-2), while two F_1_ progeny were completely excluded from the analysis (5A-1 and 11F-1). Five fermentation-related kinetic variables were derived from the weight loss data: *V*_max_, *A*_max_, length of lag phase, final weight loss, and *AF time* (Table S1). These parameters were used for QTL mapping.

LOD scores were generated from the phenotypic data from the remaining 119 F_1_ progeny, which resulted in the identification of three regions across the genome with LOD scores > 3 (see File S1). A region on chromosome III was linked to *V*_max_, whereas regions on chromosomes VII and XIII were linked to lag phase. No loci had significant linkages to *A*_max_, final weight loss, or *AF time*.

**Figure 2:**
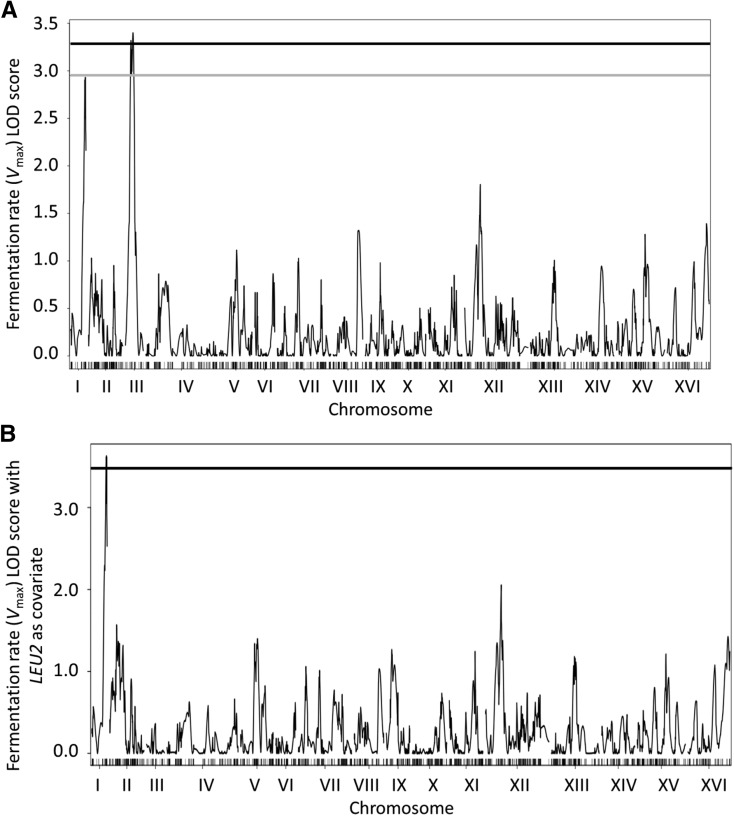
LOD scores plots of 2957 genetic markers across the 16 yeast chromosomes for *V*_max_ values across 119 BY4716 × RM11-1a F_1_ progeny. (A) LODplot including the effects of *LEU2*. The gray and black horizontal lines represent the 10 and 5% significance levels, respectively (determined from 1000 permutations of each trait). (B) LODplot using *LEU2* as covariate in a normal model to remove its effect. The black horizontal line represents the 5% significance level. LOD, Logarithm (base 10) of odds.

Closer inspection of the chromosome III region linked to *V*_max_ on SGD indicated that the linkage was due to the inclusion of the *LEU2* locus, which is deleted in RM11-1a. Removal of the effect of *LEU2* on the dataset eliminated the chromosome III peak and resulted in a significant LOD score for the QTL at the subtelomeric end of chromosome I. Figure 2 shows LOD score plots for *V*_max_ before (Figure 2A) and after (Figure 2B) the effect of *LEU2* was removed by using the *LEU2* genotype of F_1_ progeny as a covariate in a linear model of phenotype. LOD score data shows that the advantage for the *V*_max_ trait on chromosome I is derived from the BY4716 allele, and not the RM11-1a allele (File S1). This was somewhat unexpected, given that the parental fermentation data showed that S288C progressed throughout fermentation much slower than RM11-1a, although the phenomenon of “low parents” in terms of transgressive segregation has been described previously (Ehrenreich *et al.* 2009).

### Identification of two candidate genes in the chromosome I region linked to V_max_ and multiple ORFs on chromosomes VII and XIII in the regions linked to lag phase

Genes were identified in regions with LOD scores > 3 with C.I.s set at one LOD unit drop either side of a peak marker. GBrowse maps were used to identify and visualize all ORFs within the defined areas for *V*_max_ and lag phase on chromosomes I, VII, and XIII (ORFs listed in Table S2), and the presence of nucleotide differences between the parental strains was also considered as an additional criterion for candidate ORFs. Of the six ORFs in the region linked to *V*_max_ on chromosome I, two were considered to be potentially relevant to low temperature fermentation based on their respective functions: *FLO1* (*YAR050W*), encoding a cell wall lectin-like protein that binds mannose and is involved in flocculation (Miki *et al.* 1982); and *SWH1* (*YAR042W*, previously known as *OSH1*), encoding an oxysterol binding protein (Schmalix and Bandlow 1994). *S. cerevisiae swh1* mutants exhibit phenotypes similar to viable mutants defective in sterol biosynthesis and show a reduction in membrane ergosterol levels, which also results in low temperature sensitivity in a tryptophan auxotroph (Jiang *et al.* 1994; Daum *et al.* 1999). However, sequence alignment analyses using Clustal found very few allelic differences between *SWH1* in S288C and RM11-1a (99% similarity and deletion of two amino acids in S288C, see File S2). In contrast to *SWH1*, *FLO1* has a very repetitive gene structure and the allele from RM11-1a has multiple large deletions compared to S288C (see File S2). Additionally, *FLO1* is very highly expressed during fermentation at 12.5° in an F_1_ hybrid constructed by crossing another wine strain, Enoferm M2, with S288C (Deed *et al.* 2015). According to standard understanding, *FLO1* is not expressed in S288C because *FLO8*, encoding its transcriptional regulator, has a nonsense mutation and is nonfunctional; however, there are reports of *FLO1* being activated in a Flo8p-independent manner (Bester *et al.* 2006; Shen *et al.* 2006; Fichtner *et al.* 2007).

Ten ORFs were within the C.I.s near the LOD score peak for lag phase on chromosome VII (Table S2), including two genes encoding B-type cyclins involved in cell cycle progression, *CLB1* (*YGR108W*) and *CLB6* (*YGR109C*) (Surana *et al.* 1991; Schwob and Nasmyth 1993). Two neighboring peak markers with LOD scores > 3 were identified on chromosome XIII in the region linked to lag phase. Either side of these two peak markers, 34 ORFs were identified (Table S2). Genes of interest include *RCF1* (*YML030W*), encoding a cytochrome c oxidase subunit that is required for growth under hypoxic conditions (Strogolova *et al.* 2012; Vukotic *et al.* 2012), and *YOX1* (*YML027W*), encoding a transcriptional repressor involved in the regulation of cell cycle genes (Kaufmann 1993; Horak *et al.* 2002). Due to the difficulty of reproducibly phenotyping lag phase between different experiments in grape juice and the sheer number of potential candidate genes within the regions linked to lag phase, it was decided to concentrate on the identification of the locus influencing fermentation rate on chromosome I.

### The FLO1 gene is linked to V_max_

To determine the effect of the *FLO1* and *SWH1* loci on *V*_max_, deletions of *FLO1* and *SWH1* were constructed in S288C and RM11-1a and hybrids were created to perform RHA. Fermentations at 12.5 and 25° in Sauvignon Blanc juice were performed using the original S288C and RM11-1a strains (renamed S-*FS* and R-*FS* to indicate strain name and *FLO1/SWH1* genotype), the haploid *flo1* and *swh1* S288C and RM11-1a deletion strains (renamed S-*Fs*, S-*fS*, R-*Fs*, and R-*fS*), and the five diploid RHA F_1_ hybrids constructed by crossing combinations of S288C and RM11-1a wild-type, *flo1*, and *swh1* deletion strains (R-*FS* × S-*FS*, R-*Fs* × S-*FS*, R-*fS* × S-*FS*, R-*FS* × S-*Fs*, and R-*FS* × S-*fS*, see Table 2).

**Figure 3:**
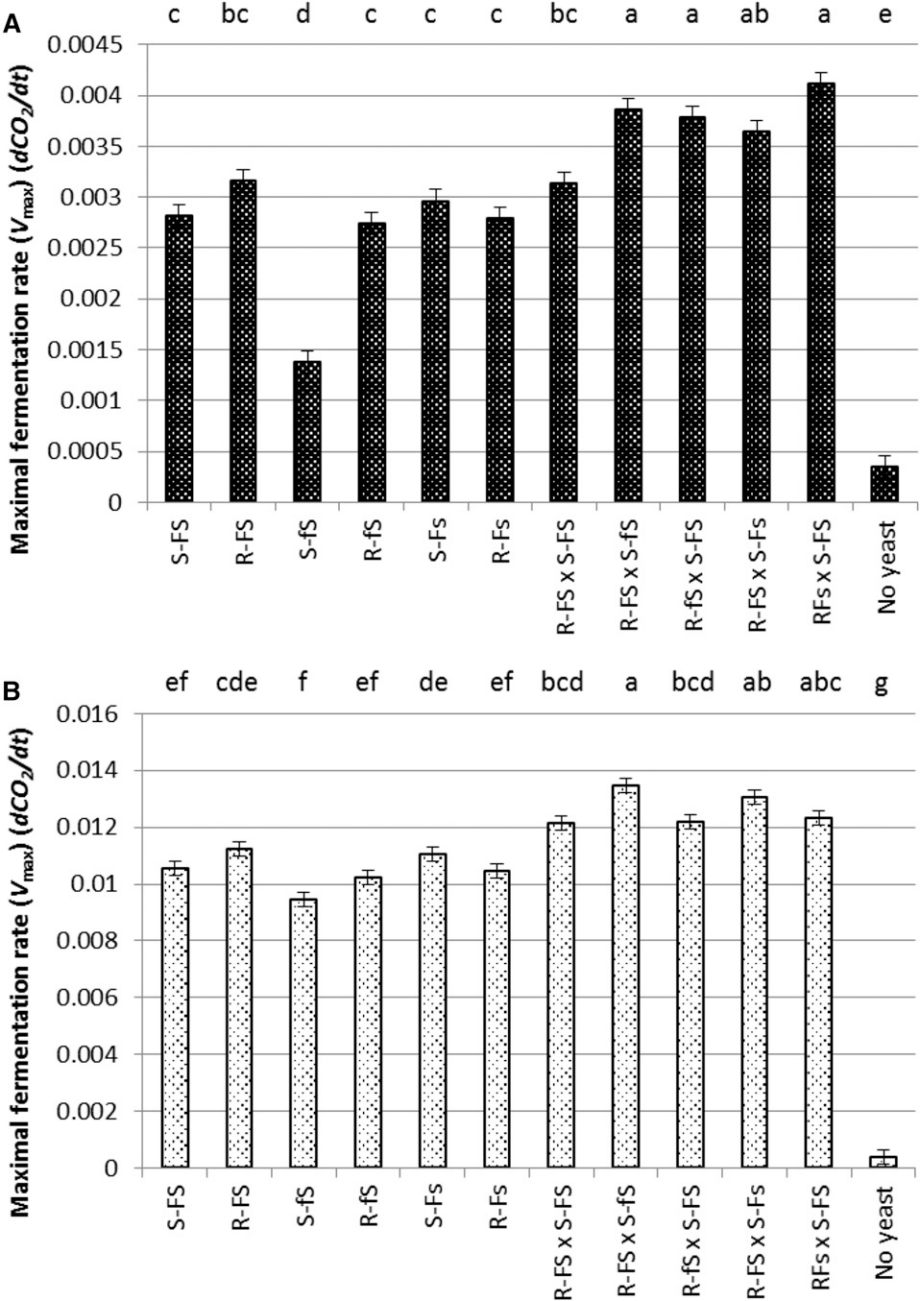
Maximal fermentation rates (*V*_max_) (*dCO_2_/dt*) of S288C (S-*FS*), RM11-1a (R-*FS*), S288C, and RM11-1a *flo1* and *swh1* gene knockouts (S-*fS*, R-*fS*, S-*Fs*, and R-*Fs*), and five F_1_ hybrids for RHA (reciprocal hemizygosity analysis) (R-*FS* × S-*FS*, R-*FS* × S-*fS*, R-*fS* × S-*FS*, R-*FS* × S-*Fs*, and R-*Fs* × S-*FS*) in Sauvignon Blanc juice. (A) 12.5°. (B) 25°. Significant differences were identified using Tukey’s HSD (honest significant difference); samples not connected by the same letter, as displayed at the top of each graph, are significantly different; *n* = 9.

*V*_max_ data are presented in Figure 3. At 12.5° (Figure 3A), there was no significant difference between the fermentation rates of S-*FS* or R-*FS* compared to three of the deletion mutants: S-*Fs*, R-*Fs*, or R-*fS*. However, the *V*_max_ of the S288C *flo1* mutant, S-*fS*, was reduced by ∼50% compared to the wild-type S-*FS* strain and the other deletion mutants. This result indicates that the *FLO1* allele is important for low temperature fermentation in S288C, but not in RM11-1a. There was no difference in *V*_max_ between the R-*FS* × S-*FS* F_1_ hybrid and the original parent strains; however, R-*FS* × S-*FS* had a slightly but significantly lower *V*_max_ than three of the RHA F_1_ hybrids: R-*Fs* × S-*FS*, R-*fS* × S-*FS*, and R-*FS* × S-*fS*. The significance of this result is not clear, but may involve uncharacterized *cis* or *trans* effects in the different strain background.

At 25° (Figure 3B), there was no significant difference between the fermentation rates of S288C (S-*FS*) and its *flo1* or *swh1* mutants (S-*fS* or S-*Fs*). There was also no difference between RM11-1a (R-*FS*) compared to its two deletion mutants, R-*fS* or R-*Fs*. However, the *V*_max_ of the S288C *swh1* mutant, S-*Fs*, was slightly but significantly higher than that of S-*Fs*. These data suggest that the deletion of the *flo1* locus does not have a significant effect on the maximal fermentation rate at higher temperature. The five diploid RHA hybrids showed only minor differences in fermentation rate, with no clear pattern emerging.

The reduction in maximal fermentation rate in the S288C *flo1* deletion strain, S-*fS*, strongly suggests that *FLO1* is linked to low temperature fermentation and most likely corresponds to the high LOD score region on chromosome I. However, RHA between S288C and RM11-1a *FLO1* gene variants did not identify any easily explained effects on maximal fermentation rate in the cold. In particular, the R-*FS* × S-*fS* F_1_ hybrid did not ferment poorly compared to the other hybrids, which would be expected if the presence of the S288C *FLO1* allele was promoting a more rapid fermentation at 12.5°.

## Discussion

Genetic linkage analysis, using a set of completely mapped 119 BY4716 × RM11-1a F_1_ progeny, identified a strong linkage between maximal fermentation rate at low temperature, and the *FLO1* gene on chromosome I. Mapping data indicated that the beneficial allele was derived from the “low parent” BY4716 and not from RM11-1a. The linkage of the BY4716 variant of *FLO1* to *V*_max_ was validated based on a 50% reduction in *V*_max_ in a cold-fermented S288C *flo1* mutant; as expected, there was no difference in *V*_max_ between the RM11-1a *flo1* deletion strain compared to RM11-1a. However, RHA between S288C and RM11-1a *FLO1* alleles did not confirm the prediction that the S288C version of *FLO1* was promoting more rapid fermentation in a different strain background.

### FLO1 has a role in stress tolerance during low temperature fermentation

The 4.6 kb *FLO1* gene encodes a cell wall surface protein that aggregates cells into “flocs” by binding to mannose sugar chains on the surfaces of other cells (Miki *et al.* 1982; Teunissen and Steensma 1995), and on substrates during glucose starvation (Fichtner *et al.* 2007). *FLO1* is one of four subtelomeric and structurally similar *FLO* genes possessed by *S. cerevisiae* (the others are *FLO5*, *FLO9*, and *FLO10*), and together they control the flocculation phenotype of different *S. cerevisiae* strains (Teunissen and Steensma 1995). Previous studies strongly suggest that the floc formation by *S. cerevisiae* is a protective mechanism against environmental and nutritional stress, since flocculation is typically induced in response to high ethanol, antifungal agents (Teunissen and Steensma 1995; Smukalla *et al.* 2008; Beauvais *et al.* 2009), and nutrient limitation [particularly carbon and/or nitrogen, see Rose (1984), Sampermans *et al.* (2005) and Stratford (1992)]. *FLO1*-expressing industrial strains also have improved fermentation performance under acetic acid stress compared to strains not expressing *FLO1* (Du *et al.* 2015), and consume hexose sugars more efficiently than nonexpressing strains in the presence of fermentation inhibitors (Westman *et al.* 2014). The subtelomeric location of *FLO1* is also in agreement with the observation that an especially high proportion of variable genes located at chromosomal telomeres are involved in fermentation (Argueso *et al.* 2009; Cubillos *et al.* 2011). The protection provided by the formation of flocs is not only due to the physical shielding of the cells in the center of the floc, but also due to an increased overall resistance to stress (Smukalla *et al.* 2008). We hypothesize that the induction of the transcriptional flocculation response not only has a role in protecting cells from chemical stressors, but also plays a role during low temperature fermentation. Smukalla *et al.* (2008) have shown that S288C cells engineered to express a Flo1+ flocculation phenotype also upregulate genes involved in cell wall, lipid, and sterol metabolism, which are also induced during the stress response to low temperatures (Beney *et al.* 2001; Gasch and Werner-Washburne 2002; Beltran *et al.* 2008; Redón *et al.* 2011; Deed *et al.* 2015). Additionally, genes within the *DAN/TIR* and *PAU* gene families, which have long been associated with the transcriptional response of *S. cerevisiae* to low temperature (Kondo and Inouye 1991; Abramova *et al.* 2001; Homma *et al.* 2003; Schade *et al.* 2004), including during fermentation at low temperatures (Beltran *et al.* 2006; Deed *et al.* 2015), are also induced in flocculating cells (Smukalla *et al.* 2008). Low fermentation temperatures may also favor flocculation due to reduced turbulence from the lower metabolic rate and slower CO_2_ formation (Soares 2011). *FLO1*-expressing cells preferentially stick to one another, regardless of genetic relatedness across the rest of the genome, suggesting a level of cooperativeness (Smukalla *et al.* 2008). This cooperation toward other cells expressing the same gene suggests that *FLO1* is one of very few “green beard genes” for altruistic social interactions. Rossouw *et al.* (2015) have taken this idea one step further by showing that *FLO* genes allow *S. cerevisiae* to form large ecological networks with non-*Saccharomyces* species, including both flocculant and nonflocculant strains. Additionally, different members of the *FLO* gene family either promote or repress certain combinations of mixed species and/or strain adhesion.

The positive influence of the S288C *FLO1* allele has never before been described for fermentation rate and this effect appeared to be enhanced significantly at low temperature (see Figure 3A). It is widely assumed that *FLO1* in S288C is not expressed, because its transcriptional regulator, Flo8p, is nonfunctional in S288C due to a nonsense mutation (Liu *et al.* 1996). In RM11-1a, the *FLO8* gene is functional and Brem *et al.* (2002) found that one-quarter of the F_1_ progeny from the BY4716 × RM11-1a cross showed a flocculation phenotype (Flo1+ and Flo8+). Gene expression microarray data from Deed *et al.* (2015) show that *FLO1* transcripts in a M2 × S288C F_1_ hybrid are dramatically upregulated during low temperature fermentation compared to the M2 parental reference. *FLO1* was upregulated 73-fold at early fermentation (2% weight loss) and 182-fold at midlate fermentation (70% weight loss). Typically, *FLO1*-dependent flocculation requires activation by Flo8p, in conjunction with another transcription factor, Mss11p, which also coregulate the *MUC1/FLO11* flocculin (Kobayashi *et al.* 1996; Bester *et al.* 2006; Fichtner *et al.* 2007). However, there are reports of *FLO1* being activated in a Flo8p-independent manner. For example, the overexpression of *MSS11*, encoding a transcription factor, can overcome the *flo8* deletion in S288C (Bester *et al.* 2006). There is evidence that *MUC1/FLO11* and *MSS11* have temperature-dependent regulation and can only facilitate trait expression at lower temperatures, strengthening the case for temperature-specific roles for cell surface proteins such as Flo1p (Lee *et al.* 2016; Taylor *et al.* 2016). Additionally, Shen *et al.* (2006) found that another transcription factor, Gts1p, could induce *FLO1* in a *flo8* mutant strain of W303-1A by binding to the Sfl1p repressor. This research supports the idea that specific environmental signals, initiating a stress response, may allow for *FLO1* to be induced in S288C, independent of Flo8p, via other transcriptional regulators. Since Flo1p tends to support cell–substrate interactions under specific environmental conditions (Fichtner *et al.* 2007), the fermentation environment may induce *FLO1* in a Flo8p-independent manner, resulting in cell–substrate adhesion, rather than flocculation *per se*. If this is the case, the ability of the S288C *FLO1*::*KanMX* (S-*fS*) mutant to form attachments to substrates within the grape solids could be visualized *vs.* the wild type using a technique such as atomic force microscopy (Canetta *et al.* 2006). Increased adhesion of yeast cells to substrates such as nutrients or grape solids may result in a higher fermentation rate at low temperature, as shown by cells that ferment while immobilized onto supports made of cellulose, gluten, corn starch, or wheat grains, in numerous studies (Mallouchos *et al.* 2003, 2007; Kandylis *et al.* 2008, 2010; Lainioti *et al.* 2011). This finding is in line with current literature demonstrating a role of Flo1p in protecting yeast from environmental stresses and improving fermentation performance under industrial conditions, including improved resistance to ethanol, acetic acid, and antimicrobial compounds (Queller 2008; Smukalla *et al.* 2008; Soares 2011; Westman *et al.* 2014; Du *et al.* 2015; Rossouw *et al.* 2015; Cheng *et al.* 2016).

The positive effect on *V*_max_ was not visible in the RHA F_1_ hybrids constructed from crosses between RM11-1a and S288C parent and deletion strains. The R-*FS* × S-*fS* RHA strain contained one *FLO1* allele from RM11-1a and the *flo1* deletion from S288C. The absence of the S288C *FLO1* allele was predicted to result in a lower *V*_max_ compared to the other RHA hybrids that possessed the wild-type version of this same allele. However, there were no significant differences between the RHA hybrids. One possible explanation is that the positive influence on *V*_max_ only occurs in haploid strains and not in diploids. The RHA F_1_ hybrids were the only diploid strains analyzed, since the two parents and all the 119 F_1_ progeny were haploid. Fichtner *et al.* (2007) state that *FLO1*-dependent flocculation is haploid-specific and that diploids display invasive or pseudohyphal growth via a nonsubtelomeric *FLO* gene, *MUC1*/*FLO11*, encoding a GPI-anchored cell surface glycoprotein required for pseudohyphal formation (Kobayashi *et al.* 1999; Guo *et al.* 2000). Haploids and diploids often differ in their tolerance to stress, even with the same genetic backgrounds, which extends to fermentation-related stressors such as ethanol (Katou *et al.* 2008; Li *et al.* 2010). Since the induction of flocculation has an impact on the ethanol resistance of *S. cerevisiae*, perhaps the influence of *FLO1* on *V*_max_ is haploid-specific and works by providing additional ethanol tolerance.

As discussed in Sinha *et al.* (2006), QTL architecture can be very complex. In this case, there may be the requirement for a second gene modifier compensator (X^R^/X^S^) that works along with *FLO1* in order for the benefit for *V*_max_ to be present. For instance, a second gene modifier may be *FLO8*, encoding the transcriptional inducer of *FLO1*; *MUC1/FLO11*; another member of the *FLO* gene family with high sequence homology, such as *FLO5*, *FLO9*, or *FLO10*; or one of three pseudogenes, *YAL065C*, *YAR061W* (merged with *YAR062W*), or *YHR213W*, with sequence similarity to other flocculin genes (Teunissen and Steensma 1995). If the advantage of S288C *FLO1* for improved fermentation performance at low temperature is Flo8p-independent, perhaps the complementation of *FLO8* via hybridization of S288C with RM11-1a prevented the S288C version of *FLO1* from having any effect on maximal fermentation rate. However, analyses of the LOD score data for *FLO8*, *MUC1/FLO11*, and the six *FLO* homologs did not identify any significant peaks corresponding to *V*_max_. *FLO* genes are particularly difficult to work with due to their highly repetitive nature and tandem repeats, high sequence homology, complex patterns of regulation, and high genetic instability (Stratford 1994; Bidard *et al.* 1995; Sato *et al.* 2001; Verstrepen *et al.* 2003; Fichtner *et al.* 2007; Liu *et al.* 2009; Van Mulders *et al.* 2010; Yue *et al.* 2013). Flocculation phenotypes also differ immensely between strains (Govender *et al.* 2008, 2010). Further research that could be performed to determine why the RHA strains had no significant differences in fermentation rate include sporulating the R-*FS* × S-*fS* RHA hybrid and measuring the maximal fermentation rate at low temperature of segregating F_1_ progeny containing the S288C *flo1* deletion. Additionally, *FLO5*, *FLO9*, and *FLO10* could be deleted in S288C to see whether these loci influence *V*_max_ at low temperature. Potential differences between the maximal fermentation rate of *flo1* haploids and diploids could also be investigated.

### Conclusions

We have identified a QTL linked to *V*_max_ and two QTL linked to lag phase in *S. cerevisiae*. Deletion of candidate genes confirmed that the gene on chromosome I linked to *V*_max_ in S288C is *FLO1*, encoding a yeast flocculin. Deletion of *FLO1* in the haploid S288C strain resulted in a large decrease in fermentation rate at 12.5°, but no change at 25°. A greater understanding of the role of the *FLO* family in stress tolerance will allow easier manipulation and/or selection of *S. cerevisiae* strains to improve *V*_max_ and provide growth advantages during the low temperature fermentation of foods and beverages.

## Supplementary Material

Supplemental material is available online at www.g3journal.org/lookup/suppl/doi:10.1534/g3.116.037630/-/DC1.
