# Supplementary material for: Saccharomyces cerevisiae FLO1 Gene Demonstrates Genetic Linkage to Increased Fermentation Rate at Low Temperatures
Source: G3 (Bethesda). 2017 Jan 30;7(3):1039–48. doi: 10.1534/g3.116.037630 (PMC5345705; doi:10.1534/g3.116.037630)
Supplement: Supplementary file 1 [file 1039TableS1.docx]

**Table S1** Fermentation parameters (*V*_max_, *A*_max_, lag phase, final weight loss and *AF time*) of the BY4716 × RM11-1a F_1_ progeny and parental strains for linkage analysis. F_1_ progeny names were derived from 96-well plate position, with the suffix -2 denoting the second plate

| Name | YLK name | Vial name | *V*_max_  (*dCO_2_/dt*) | *A*_max_ (*d^2^CO_2_/dt^2^*) | Lag phase (h) | Final weight loss (g) | *AF time* (h) |
| --- | --- | --- | --- | --- | --- | --- | --- |
| 1A | YLK103 | 1_1_d | 0.0036 | 5.96E-05 | 150 | 0.670 | 420 |
| 1B | YLK145 | 2_7_b | 0.0040 | 5.84E-05 | 120 | 0.758 | 480 |
| 1C | YLK187 | 5_1_d | 0.0051 | 8.85E-05 | 75 | 0.827 | 445 |
| 1D | YLK235 | 7_1_d | 0.0047 | 6.71E-05 | 95 | 0.763 | 505 |
| 1E | YLK281 | 8_5_b | 0.0049 | 7.00E-05 | 80 | 0.811 | 395 |
| 1F | YLK335 | 10_4_d | 0.0035 | 5.47E-05 | 75 | 0.778 | 495 |
| 1G | YLK377 | 14_1_b | 0.0048 | 7.47E-05 | 80 | 0.843 | 515 |
| 1H | YLK427 | 16_1_d | 0.0049 | 8.16E-05 | 90 | 0.814 | 480 |
| 2A | YLK107 | 1_2_d | 0.0036 | 4.11E-05 | 90 | 0.797 | 510 |
| 2B | YLK146 | 2_7_c | 0.0050 | 8.10E-05 | 115 | 0.829 | 360 |
| 2C | YLK191 | 5_2_d | 0.0046 | 6.89E-05 | 90 | 0.828 | 430 |
| 2D | YLK238 | 7_2_c | 0.0054 | 8.51E-05 | 85 | 0.830 | 340 |
| 2E | YLK286 | 8_6_c | 0.0051 | 6.66E-05 | 95 | 0.834 | 405 |
| 2F | YLK336 | 11_1_a | 0.0047 | 9.90E-05 | 95 | 0.853 | 485 |
| 2G | YLK382 | 14_2_c | 0.0046 | 8.59E-05 | 75 | 0.852 | 495 |
| 2H | YLK428 | 17_1_a | 0.0058 | 1.02E-04 | 90 | 0.866 | 360 |
| 3A | YLK111 | 1_3_d | 0.0047 | 6.83E-05 | 90 | 0.833 | 480 |
| 3B | YLK147 | 2_7_d | 0.0047 | 6.08E-05 | 110 | 0.856 | 435 |
| 3C | YLK195 | 5_3_d | 0.0048 | 8.68E-05 | 125 | 0.832 | 375 |
| 3D | YLK243 | 7_3_d | 0.0055 | 9.64E-05 | 100 | 0.832 | 420 |
| 3E | YLK289 | 8_7_b | 0.0044 | 5.27E-05 | 90 | 0.816 | 430 |
| 3F | YLK343 | 11_2_d | 0.0039 | 4.95E-05 | 100 | 0.781 | 470 |
| 3G | YLK387 | 14_3_d | 0.0041 | 6.66E-05 | 90 | 0.762 | 480 |
| 3H | YLK435 | 17_2_d | 0.0057 | 9.72E-05 | 90 | 0.833 | 360 |
| 4A | YLK115 | 1_4_d | 0.0049 | 8.33E-05 | 85 | 0.818 | 415 |
| 4B | YLK151 | 3_1_d | 0.0043 | 5.09E-05 | 85 | 0.819 | 415 |
| 4C | YLK199 | 5_4_d | 0.0041 | 1.07E-04 | 60 | 0.818 | 535 |
| 4D | YLK246 | 7_4_c | 0.0048 | 7.29E-05 | 105 | 0.827 | 465 |
| 4E | YLK295 | 9_1_d | 0.0051 | 1.15E-04 | 70 | 0.849 | 450 |
| 4G | YLK388 | 14_4_a | 0.0051 | 9.90E-05 | 90 | 0.850 | 410 |
| 4H | YLK436 | 17_4_a | 0.0060 | 1.00E-04 | 85 | 0.856 | 295 |
| 5B | YLK155 | 3_2_d | 0.0042 | 9.77E-05 | 100 | 0.826 | 490 |
| 5C | YLK203 | 5_5_d | 0.0048 | 8.10E-05 | 90 | 0.820 | 480 |
| 5D | YLK251 | 7_5_d | 0.0047 | 7.15E-05 | 85 | 0.851 | 460 |
| 5E | YLK299 | 9_2_d | 0.0045 | 6.54E-05 | 95 | 0.815 | 495 |
| 5F | YLK345 | 11_3_b | 0.0055 | 8.59E-05 | 65 | 0.847 | 395 |
| 5G | YLK393 | 14_5_b | 0.0047 | 7.38E-05 | 80 | 0.844 | 465 |
| 5H | YLK441 | 17_5_b | 0.0050 | 8.45E-05 | 90 | 0.842 | 405 |
| 6A | YLK118 | 1_5_c | 0.0057 | 1.02E-04 | 90 | 0.874 | 420 |
| 6B | YLK159 | 3_3_d | 0.0045 | 6.51E-05 | 80 | 0.791 | 510 |
| 6C | YLK207 | 6_1_d | 0.0035 | 4.75E-05 | 100 | 0.744 | 300 |
| 6D | YLK254 | 7_6_c | 0.0061 | 8.45E-05 | 100 | 0.845 | 490 |
| 6E | YLK303 | 9_3_d | 0.0042 | 6.90E-05 | 50 | 0.809 | 570 |
| 6F | YLK351 | 12_1_d | 0.0039 | 6.25E-05 | 90 | 0.806 | 500 |
| 6G | YLK399 | 14_6_d | 0.0041 | 7.18E-05 | 90 | 0.783 | 480 |
| 6H | YLK447 | 18_1_d | 0.0037 | 6.51E-05 | 75 | 0.795 | 495 |
| 7A | YLK127 | 2_2_d | 0.0058 | 8.97E-05 | 110 | 0.822 | 390 |
| 7B | YLK163 | 3_4_d | 0.0043 | 7.52E-05 | 85 | 0.837 | 415 |
| 7C | YLK209 | 6_2_b | 0.0032 | 4.51E-05 | 105 | 0.704 | 445 |
| 7D | YLK258 | 7_7_c | 0.0051 | 8.51E-05 | 90 | 0.824 | 480 |
| 7E | YLK307 | 9_4_d | 0.0036 | 3.94E-05 | 85 | 0.771 | 535 |
| 7F | YLK353 | 12_2_b | 0.0043 | 6.42E-05 | 90 | 0.804 | 480 |
| 7G | YLK402 | 14_7_c | 0.0040 | 1.17E-04 | 65 | 0.792 | 480 |
| 7H | YLK451 | 18_2_d | 0.0049 | 6.13E-05 | 90 | 0.830 | 430 |
| 8A | YLK131 | 2_3_d | 0.0047 | 8.04E-05 | 95 | 0.843 | 450 |
| 8B | YLK167 | 3_5_d | 0.0039 | 6.60E-05 | 85 | 0.815 | 485 |
| 8C | YLK214 | 6_3_c | 0.0050 | 1.07E-04 | 65 | 0.852 | 505 |
| 8D | YLK263 | 7_8_d | 0.0050 | 9.26E-05 | 90 | 0.861 | 410 |
| 8E | YLK311 | 9_5_d | 0.0049 | 8.39E-05 | 120 | 0.819 | 430 |
| 8F | YLK356 | 13_1_a | 0.0031 | 4.98E-05 | 95 | 0.732 | 475 |
| 8G | YLK407 | 15_2_d | 0.0046 | 7.38E-05 | 95 | 0.727 | 425 |
| 8H | YLK455 | 18_3_d | 0.0044 | 7.12E-05 | 85 | 0.825 | 455 |
| 9A | YLK132 | 2_4_a | 0.0052 | 9.20E-05 | 90 | 0.855 | 480 |
| 9B | YLK170 | 4_1_c | 0.0049 | 7.93E-05 | 95 | 0.857 | 425 |
| 9C | YLK219 | 6_4_d | 0.0051 | 6.77E-05 | 70 | 0.857 | 450 |
| 9D | YLK264 | 8_1_a | 0.0051 | 7.58E-05 | 115 | 0.842 | 430 |
| 9E | YLK315 | 9_6_d | 0.0048 | 7.18E-05 | 120 | 0.813 | 425 |
| 9F | YLK362 | 13_2_c | 0.0049 | 1.19E-04 | 130 | 0.854 | 390 |
| 9G | YLK409 | 15_3_b | 0.0058 | 8.45E-05 | 85 | 0.844 | 365 |
| 9H | YLK458 | 18_4_c | 0.0048 | 7.12E-05 | 90 | 0.849 | 430 |
| 10A | YLK139 | 2_5_d | 0.0047 | 7.52E-05 | 90 | 0.836 | 500 |
| 10B | YLK172 | 4_2_a | 0.0033 | 4.98E-05 | 70 | 0.768 | 480 |
| 10C | YLK223 | 6_5_d | 0.0042 | 5.56E-05 | 90 | 0.799 | 480 |
| 10D | YLK271 | 8_2_d | 0.0050 | 8.10E-05 | 115 | 0.858 | 405 |
| 10E | YLK319 | 9_7_d | 0.0045 | 6.66E-05 | 115 | 0.830 | 450 |
| 10F | YLK365 | 13_3_b | 0.0047 | 6.89E-05 | 110 | 0.851 | 460 |
| 10G | YLK415 | 15_4_d | 0.0043 | 4.34E-05 | 95 | 0.842 | 495 |
| 10H | YLK463 | 18_6_d | 0.0043 | 5.79E-05 | 105 | 0.841 | 465 |
| 11A | YLK143 | 2_6_d | 0.0039 | 5.32E-05 | 100 | 0.840 | 420 |
| 11B | YLK179 | 4_3_d | 0.0044 | 6.02E-05 | 130 | 0.846 | 415 |
| 11C | YLK227 | 6_6_d | 0.0048 | 8.56E-05 | 90 | 0.850 | 430 |
| 11D | YLK272 | 8_3_a | 0.0054 | 8.22E-05 | 95 | 0.877 | 355 |
| 11E | YLK322 | 10_1_c | 0.0032 | 3.65E-05 | 125 | 0.728 | 445 |
| 11G | YLK417 | 15_5_b | 0.0039 | 7.64E-05 | 90 | 0.780 | 480 |
| 12A | YLK144 | 2_7_a | 0.0044 | 6.37E-05 | 90 | 0.824 | 480 |
| 12B | YLK183 | 4_4_d | 0.0038 | 4.80E-05 | 105 | 0.794 | 485 |
| 12C | YLK231 | 6_7_d | 0.0042 | 7.75E-05 | 90 | 0.853 | 500 |
| 12D | YLK278 | 8_4_c | 0.0041 | 5.79E-05 | 95 | 0.824 | 475 |
| 12E | YLK327 | 10_2_d | 0.0053 | 7.55E-05 | 65 | 0.849 | 455 |
| 12F | YLK374 | 13_5_c | 0.0045 | 8.39E-05 | 85 | 0.826 | 485 |
| 12G | YLK422 | 15_6_c | 0.0041 | 7.03E-05 | 45 | 0.858 | 475 |
| 1A-2 | YLK466 | 19_1_c | 0.0044 | 8.56E-05 | 85 | 0.836 | 485 |
| 1B-2 | YLK515 | 21_3_d | 0.0053 | 8.62E-05 | 95 | 0.850 | 425 |
| 1C-2 | YLK571 | 25_3_d | 0.0047 | 8.51E-05 | 100 | 0.854 | 470 |
| 2A-2 | YLK470 | 19_2_c | 0.0042 | 6.08E-05 | 90 | 0.768 | 455 |
| 2B-2 | YLK519 | 21_4_d | 0.0051 | 9.09E-05 | 95 | 0.835 | 405 |
| 2C-2 | YLK575 | 25_4_d | 0.0043 | 7.12E-05 | 80 | 0.795 | 490 |
| 3A-2 | YLK474 | 19_3_c | 0.0041 | 8.20E-05 | 75 | 0.797 | 545 |
| 3B-2 | YLK522 | 21_5_c | 0.0053 | 9.32E-05 | 85 | 0.848 | 415 |
| 3C-2 | YLK583 | 26_1_d | 0.0043 | 8.80E-05 | 80 | 0.807 | 515 |
| 4A-2 | YLK477 | 19_4_b | 0.0049 | 6.94E-05 | 110 | 0.829 | 460 |
| 4B-2 | YLK527 | 22_1_d | 0.0052 | 6.71E-05 | 75 | 0.855 | 375 |
| 4C-2 | YLK587 | 26_2_d | 0.0045 | 7.55E-05 | 70 | 0.806 | 470 |
| 5A-2 | YLK481 | 19_5_b | 0.0046 | 7.12E-05 | 90 | 0.830 | 430 |
| 5B-2 | YLK531 | 22_2_d | 0.0040 | 5.50E-05 | 100 | 0.840 | 420 |
| 5C-2 | YLK591 | 26_3_d | 0.0044 | 7.81E-05 | 90 | 0.850 | 430 |
| 6A-2 | YLK487 | 20_1_d | 0.0036 | 4.28E-05 | 70 | 0.763 | 450 |
| 6B-2 | YLK533 | 22_3_b | 0.0042 | 6.37E-05 | 80 | 0.797 | 510 |
| 7A-2 | YLK491 | 20_2_d | 0.0047 | 7.93E-05 | 75 | 0.866 | 425 |
| 7B-2 | YLK539 | 22_4_d | 0.0051 | 5.96E-05 | 80 | 0.873 | 390 |
| 8A-2 | YLK494 | 20_3_c | 0.0049 | 6.66E-05 | 105 | 0.853 | 395 |
| 8B-2 | YLK543 | 22_5_d | 0.0044 | 7.29E-05 | 145 | 0.819 | 455 |
| 9A-2 | YLK498 | 20_4_c | 0.0050 | 7.23E-05 | 130 | 0.788 | 440 |
| 9B-2 | YLK551 | 23_3_d | 0.0052 | 5.73E-05 | 95 | 0.856 | 305 |
| 9C-2 | YLK330 | 10_3_c | 0.0053 | 6.42E-05 | 105 | 0.861 | 345 |
| 10A-2 | YLK503 | 20_5_d | 0.0040 | 5.38E-05 | 105 | 0.794 | 465 |
| 10B-2 | YLK559 | 23_5_d | 0.0037 | 7.81E-05 | 100 | 0.811 | 490 |
| 11A-2 | YLK507 | 21_1_d | 0.0031 | 4.43E-05 | 105 | 0.804 | 485 |
| 11B-2 | YLK563 | 24_1_d | 0.0041 | 4.80E-05 | 105 | 0.837 | 465 |
| 12A-2 | YLK511 | 21_2_d | 0.0037 | 5.38E-05 | 105 | 0.845 | 465 |
| 12B-2 | YLK567 | 25_1_d | 0.0044 | 7.06E-05 | 85 | 0.850 | 435 |
| RM11-1a | - | - | 0.0037 | 6.60E-05 | 85 | 0.835 | 460 |
| S288C | - | - | 0.0021 | 3.91E-05 | 60 | 0.672 | 560 |
