# Supplementary material for: Saccharomyces cerevisiae FLO1 Gene Demonstrates Genetic Linkage to Increased Fermentation Rate at Low Temperatures
Source: G3 (Bethesda). 2017 Jan 30;7(3):1039–48. doi: 10.1534/g3.116.037630 (PMC5345705; doi:10.1534/g3.116.037630)
Supplement: Supplementary file 2 [file 1039TableS2.docx]

**Table S2** List of ORFs identified within one LOD unit either side of the LOD > 3 peak markers for traits linked to fermentation kinetics in the *S. cerevisiae* genome. Marker peaks are highlighted in bold and the descriptions of function were obtained from SGD

| Chromosome | Trait | LOD score | ORF | Gene | Function |
| --- | --- | --- | --- | --- | --- |
| I | *V*_max_ | 2.863 | *YAR035W* | *YAT1* | Outer mitochondrial carnitine acetyltransferase |
| I | *V*_max_ | 2.863 | *YAR035C-A* | *NA* | Putative protein of unknown function |
| I | *V*_max_ | 2.863 | *YAR042W* | *SWH1* | Similar to mammalian oxysterol-binding protein |
| I | *V*_max_ | 3.630-**3.619** | *YAR047C* | *NA* | Dubious open reading frame |
| I | *V*_max_ | **3.619**-3.037 | *YAR050W* | *FLO1* | Lectin-like protein involved in flocculation |
| I | *V*_max_ | 2.921 | *YAR053W* | *NA* | Dubious open reading frame |
| VII | Lag phase | 2.065-2.292 | *YGR103W* | *NOP7* | Component of several different pre-ribosomal particles |
| VII | Lag phase | 2.235-2.570 | *YGR104C* | *SRB5* | Subunit of the RNA polymerase II mediator complex |
| VII | Lag phase | 2.642-**3.000** | *YGR105W* | *VMA21* | Integral membrane protein required for V-ATPase function |
| VII | Lag phase | 2.642-**3.000** | *YGR106C* | *VOA1* | ER protein that functions in assembly of the V0 sector of V-ATPase |
| VII | Lag phase | 2.642-**3.000** | *YGR107W* | *NA* | Dubious open reading frame |
| VII | Lag phase | 2.642-**3.000** | *YGR108W* | *CLB1* | B-type cyclin involved in cell cycle progression |
| VII | Lag phase | 2.978 | *YGR109C* | *CLB6* | B-type cyclin involved in DNA replication during S phase |
| VII | Lag phase | 2.979-2.030 | *YGR109W-A* | *NA* | Retrotransposon TYA Gag gene co-transcribed with TYB Pol |
| VII | Lag phase | 2.979-2.030 | *YGR109W-B* | *NA* | Retrotransposon TYA Gag and TYB Pol genes |
| VII | Lag phase | 2.979-2.030 | *YGR110W* | *CLD1* | Mitochondrial cardiolipin-specific phospholipase |
| XIII | Lag phase | 2.273-2.623 | *YML049C* | *RSE1* | Protein involved in pre-mRNA splicing |
| XIII | Lag phase | 2.606 | *YML048W* | *GSF2* | Endoplasmic reticulum localized integral membrane protein |
| XIII | Lag phase | 2.606-**3.175** | *YML047W-A* | *NA* | Dubious open reading frame |
| XIII | Lag phase | 2.606-**3.175** | *YML047C* | *PRM6* | Potassium transporter that mediates K^+^ influx |
| XIII | Lag phase | 2.606-**3.175** | *YML046W* | *PRP39* | U1 snRNP protein involved in splicing |
| XIII | Lag phase | 2.606-**3.175** | *YML045W* | *NA* | Retrotransposon TYA Gag and TYB Pol genes |
| XIII | Lag phase | 2.606-**3.175** | *YML045W-A* | *NA* | Retrotransposon TYA Gag gene co-transcribed with TYB Pol |
| XIII | Lag phase | 2.606-**3.175** | *YML043C* | *RRN11* | Component of the core factor rDNA transcription factor complex |
| XIII | Lag phase | 2.606-**3.175** | *YML042W* | *CAT2* | Carnitine acetyl-CoA transferase |
| XIII | Lag phase | 2.606-**3.175** | *YML041C* | *VPS71* | Nucleosome-binding component of the SWR1 complex |
| XIII | Lag phase | 2.606-**3.175** | *YML040W* | *NA* | Retrotransposon TYA Gag gene co-transcribed with TYB Pol |
| XIII | Lag phase | 2.606-**3.175** | *YML039W* | *NA* | Retrotransposon TYA Gag and TYB Pol genes |
| XIII | Lag phase | **3.175** | *YML038C* | *YMD8* | Putative nucleotide sugar transporter |
| XIII | Lag phase | 3.119-2.720 | *YML037C* | *NA* | Putative protein of unknown function |
| XIII | Lag phase | 2.478 | *YML036W* | *CGI121* | Component of the EKC/KEOPS complex |
| XIII | Lag phase | 2.547-3.681 | *YML035C* | *AMD1* | AMP deaminase |
| XIII | Lag phase | 2.547-3.681 | *YML034C-A* | *NA* | Dubious open reading frame |
| XIII | Lag phase | 2.547-3.681 | *YML034W* | *SRC1* | Inner nuclear membrane protein |
| XIII | Lag phase | 2.547-3.681 | *YML032C* | *RAD52* | Protein that stimulates strand exchange |
| XIII | Lag phase | 2.547-3.681 | *YML031C-A* | *NA* | Dubious open reading frame |
| XIII | Lag phase | 3.681 | *YML031W* | *NDC1* | Subunit of the transmembrane ring of the nuclear pore complex |
| XIII | Lag phase | **3.725-**3.373 | *YML030W* | *RCF1* | Cytochrome c oxidase subunit |
| XIII | Lag phase | **3.725-**3.373 | *YML029W* | *USA1* | Scaffold subunit of the Hrd1p ubiquitin ligase |
| XIII | Lag phase | **3.725-**3.373 | *YML028W* | *TSA1* | Thioredoxin peroxidase |
| XIII | Lag phase | **3.725-**3.373 | *YML027W* | *YOX1* | Homeobox transcriptional repressor; binds to Mcm1p and to early cell cycle boxes in the promoters of cell cycle-regulated genes expressed in M/G1 phase |
| XIII | Lag phase | **3.725-**3.373 | *YML026C* | *RPS18B* | Protein component of the small (40S) ribosomal subunit |
| XIII | Lag phase | **3.725-**3.373 | *YML025C* | *YML6* | Mitochondrial ribosomal protein of the large subunit |
| XIII | Lag phase | **3.725-**3.373 | *YML024W* | *RPS17A* | Ribosomal protein 51 (rp51) of the small (40s) subunit |
| XIII | Lag phase | 3.328 | *YML023C* | *NSE5* | Component of the SMC5-SMC6 complex |
| XIII | Lag phase | 3.328 | *YML022W* | *APT1* | Adenine phosphoribosyltransferase |
| XIII | Lag phase | 3.421-3.288 | *YML021C* | *UNG1* | Uracil-DNA glycosylase |
| XIII | Lag phase | 3.421-3.288 | *YML020W* | *NA* | Protein of unknown function |
| XIII | Lag phase | 3.421-3.288 | *YML019W* | *OST6* | Subunit of the oligosaccharyltransferase complex of the ER lumen |
| XIII | Lag phase | 3.288 | *YML018C* | *NA* | Protein of unknown function |
