# Supplementary material for: Saccharomyces cerevisiae FLO1 Gene Demonstrates Genetic Linkage to Increased Fermentation Rate at Low Temperatures
Source: G3 (Bethesda). 2017 Jan 30;7(3):1039–48. doi: 10.1534/g3.116.037630 (PMC5345705; doi:10.1534/g3.116.037630)
Supplement: Supplementary file 4 [file 1039FileS2.docx]

**File S2** *FLO1* and *SWH1* Clustal multiple sequence alignments for RM11-1a and S288C

S288C_FLO1 ------------------------------------------------------------

RM11-1a_FLO1 ATGGGAACAAAAAAATAACTCCAGTGCTGCTAATTAGACTAAAAATATGAAAAAAAAAGG 60

S288C_FLO1 ------------------------------------------------------------

RM11-1a_FLO1 AGAGGGCGAGGATGAGTATATCTAACCCACATAGCTTGAAAGTTGCTCGCTACATGCTAA 120

S288C_FLO1 ------------------------------------------------------------

RM11-1a_FLO1 AAGTTTACTCAAATTACAAATCATCACTTTCATGAACATCCTCTACTAGCTATTATGAAG 180

S288C_FLO1 ------------------------------------------------------------

RM11-1a_FLO1 GCGCAGAGAGTGGTCTCGTAACCAATTTTATGCAAGTCGTTGAAAAAACGGCGGCTCTTT 240

S288C_FLO1 ------------------------------------------------------------

RM11-1a_FLO1 AGTAGACTGGTCAAGCGGCATCGGAAACAGTTCTCAGAACAGAAAAGAAGAGATTATTGA 300

S288C_FLO1 ------------------------------------------------------------

RM11-1a_FLO1 GTTAAGGTCCATTAAAGCGTTATGATAAGCCCAATAAGATACCAAGTAGACATGTTACAC 360

S288C_FLO1 ------------------------------------------------------------

RM11-1a_FLO1 CGTGAGTAGTAAACGGGGTTATATTTTATTATGTTGCTAGTCTCATTTTATGGCACTTGT 420

S288C_FLO1 ------------------------------------------------------------

RM11-1a_FLO1 AGTTTGGAGGAAAAATGATCGGATAATCAAAACGAAATTCCTTAAGTGATTTGTGTCCCT 480

S288C_FLO1 ------------------------------------------------------------

RM11-1a_FLO1 AAAAGAAAATCTTCAGGTATAACTGCAAACGCCAAATGCCACAAGCCCAAAAATTTCCGT 540

S288C_FLO1 ------------------------------------------------------------

RM11-1a_FLO1 GTCTTTTCCCGATGCTATCCTTGATTAAGGAACTTTTGACTGTCAAAAGTGAAAATATCT 600

S288C_FLO1 ------------------------------------------------------------

RM11-1a_FLO1 ATATGGACTCTTTTGAAGAATTAAAAGAAATAGGCAAATGCCACTTGTGTACATTACACT 660

S288C_FLO1 -------------------------------------AAAAAAAAGTGCATTTATTTAGG 23

RM11-1a_FLO1 ACCTACTAAACGATTTCGTAACCTAGGCTAATACGCGAAACAAGCGTTTTTTTATTTAGT 720

*** ** ** *********

S288C_FLO1 TAAGTCTCAT--TACCTAAACGCCAGTTTGTTTCACGTAATTGGTAACGATGAGGGAACC 81

RM11-1a_FLO1 GAAGCATCATATTACCTAAGCGCCACTTTGCTTGACGTTAGTAACAAAAATGGTGAAATT 780

*** **** ******* ***** **** ** **** * * ** *** * **

S288C_FLO1 GCAGTAGAAAAAACTTTCATTCACAAACGATTAAAGTGTTATGCTAGCCAGTTTCAGGCT 141

RM11-1a_FLO1 GTAATAGAAGAAACTTTCGTCCGCAAACTATTGAGGTACTATGCTTGTCCGTCTCAAGCT 840

* * ***** ******** * * ***** *** * ** ****** * * ** *** ***

S288C_FLO1 TTTTGTTTTATGCAAGAGAACATTCGACTAGATGTCCAGTTAAGTGTGCGTCACTTTTCC 201

RM11-1a_FLO1 ----GTTTGATGCAAGAGAACGGTCAACTCAATTTCCGGCTA---GTACGTCACTTTTCC 893

**** ************ ** *** ** *** * ** ** ************

S288C_FLO1 TACGGTG-CCTCGCACATGAATGTTATCCGGCGCACGATACTTATCACCGAAAAACCTTA 260

RM11-1a_FLO1 TACGGTGGCTTCGCAGACGAATGTTTTCCGACACATGATACTTATCACCGAAAAACCTTA 953

******* * ***** * ******* **** * ** ************************

S288C_FLO1 TTCTACGGAAAA--CCTTATTTACATTAAAGTTGGAAAAATTTCCTCTTTTTCCTAATAA 318

RM11-1a_FLO1 TTTCACGGAAAAAACCTTATTTACATTAAAGTTTGAAAAATTTTCTTCTTTCCGCAATAT 1013

** ******** ******************* ********* ** *** * ****

S288C_FLO1 GGTGGAGCTTTTGGCTTCCAGTATGCTTTCACGGAATTATTTCTCATGTACATTTAGCTC 378

RM11-1a_FLO1 GGTGGGGCCTTTGACTTCCAGTATGCTTTCACGGAATTATTTCTGATGTACATTTAGCTC 1073

***** ** **** ****************************** ***************

S288C_FLO1 CATTTCCAGTGCCTCCGATAGGGAGGCATCATGGTACTACCGTGACGGAGAATACGTAGG 438

RM11-1a_FLO1 CATTTCCAGTGCCTCCGATAGGAAGGCATCATGGTACTACCGTGACAGAGAATACGTAGG 1133

********************** *********************** *************

S288C_FLO1 CTGACTTTTTCGTCAGTTTGTTGTCCGTTTACAAAATTGGTGAATGAATTCTAGCCTTCC 498

RM11-1a_FLO1 CTGACTTTTTCGTCAGTTTGTTGTCCGTTTACAAAATTGGTGAATGAATTCTAGCCTT-- 1191

**********************************************************

S288C_FLO1 TCTGCTCATTAATTGCCCTCACAAGAATTTGGAAGTGCGTAGA-CAGGTAAAAGATTGTA 557

RM11-1a_FLO1 --TGCTCATTAATTGCCCTCACAAGAATTTGGAAGTGCGTAGAACAGGTAAAAGGTTGCA 1249

***************************************** ********** *** *

S288C_FLO1 CTACAGAGGTATTGTGGAACCTTCTACAGTACTTCGGAATACACCTAAAAGGTTGTTGGA 617

RM11-1a_FLO1 CTACAGAGGTATTGTGGATCCTTCTACAGTACTTCGGAATACACCTAAAAGGTTGTTGGA 1309

****************** *****************************************

S288C_FLO1 TGCTAAATTTAGCAAAAGTCTTTTTTAGCTCACTATTAGGCTTGTTAAAGTCTGAAATTG 677

RM11-1a_FLO1 TGCTAAATTTAGCAAAAGTCTTTTTTAGCTTACTATTAGGCTTGTTAAAGTCTGAAATTG 1369

****************************** *****************************

S288C_FLO1 TTGAAAGGCACTCAAAAAGATAAATCAACAATCAGCATTAACGGCACAGTTGAAAGAGTC 737

RM11-1a_FLO1 TTGAAAGGCACTCAAAA-GATAAATCGACAATTAGCATTAACGGCACAGTTGAAAGAGTC 1428

***************** ******** ***** ***************************

S288C_FLO1 ACCCACTTGAAATTAGCTCGGTTATCAAATATAATTATCTCTGGTAAAGAGCTCTGCAGC 797

RM11-1a_FLO1 ACCCACTTGAAATTAGCTCGGTTATCAAATATAATTATCTCCAGTAAAGAGCTCTGCAGC 1488

***************************************** *****************

S288C_FLO1 AGGGTTAATCTATTCGCATACTTACGCTGTAGGAACATTTTATTATTAGGATCCGACTAC 857

RM11-1a_FLO1 AGGGTTAATCTATTCCTATACTTCCGCTGTAGGAACATTTTATTATTAGGATCCGACTAC 1548

*************** ****** ************************************

S288C_FLO1 TGCCTACATATTTATTCGGAAGGCATGATGTCGAAAATTTTTGAGCTTATAAAAGGAACA 917

RM11-1a_FLO1 TGCCTACATATTTATTCGGAAGGCTTGATGTCGAAAATTTTTAAGCTTATAAAAAGAACA 1608

************************ ***************** *********** *****

S288C_FLO1 TATTTCACTCTTGCTCGTT-GATGTAAGCTCTCTTCCGGGTTCTTATTTTTAATTCTTGT 976

RM11-1a_FLO1 TATTTCACTCTTGCTCGTTTGATGTAAGCTTTCTTCCGGGTTCTTATTTTTAATTCTTGT 1668

******************* ********** *****************************

S288C_FLO1 CACCAGTAAACAGAACATCCAAAAATGACAATGCCTCATCGCTATATGTTTTTGGCAGTC 1036

RM11-1a_FLO1 CACCAGTTAACAGAACATCCAAAAATGACAATGCCTCATCGCTATATGTTTTTGGCAGTC 1728

******* ****************************************************

S288C_FLO1 TTTACACTTCTGGCACTAACTAGTGTGGCCTCAGGAGCCACAGAGGCGTGCTTACCAGCA 1096

RM11-1a_FLO1 TTTACACTTCTGGCAGTGATTAATGTGGCCTCAGGAGCCACAGAGGCGTGCTTACCAGCA 1788

*************** * * ** *************************************

S288C_FLO1 GGCCAGAGGAAAAGTGGGATGAATATAAATTTTTACCAGTATTCATTGAAAGATTCCTCC 1156

RM11-1a_FLO1 GGCCAGAGGAAAAGTGGGATGAATATAAATTTTTACCAGTATTCATTGAAAGATTCCTCC 1848

************************************************************

S288C_FLO1 ACATATTCGAATGCAGCATATATGGCTTATGGATATGCCTCAAAAACCAAACTAGGTTCT 1216

RM11-1a_FLO1 ACGTATTCTAATGCAGCATATATGGCTTACCAATATGCAGACAAAGTCAAATTGGGCTCT 1908

** ***** ******************** ****** *** **** * ** ***

S288C_FLO1 GTCGGAGGACAAACTGATATCTCGATTGATTATAATATTCCCTGTGTTAGTTCATCAGGC 1276

RM11-1a_FLO1 GTTAGTGGGCAAACGGATATATCTATCAACTATAATCTTCCTTGTGTTACAACCTCAGGG 1968

** * ** ***** ***** ** ** * ****** **** ******* * *****

S288C_FLO1 ACATTTCCTTGTCCTCAAGAAGATTCCTATGGAAACTGGGGATGCAAAGGAATGGGTGCT 1336

RM11-1a_FLO1 ACATATCAGTGCCCTCAAGAAGATGCATATGGTAATTGGGGATGCAGAGGTAAGGGGAGA 2028

**** ** ** ************ * ***** ** ********** *** * ***

S288C_FLO1 TGTTCTAATAGTCAAGGAATTGCATACTGGAGTACTGATTTATTTGGTTTCTATACTACC 1396

RM11-1a_FLO1 TGCTCCAACAGTCAAGCAGTTTCATACTGGAGTACAGATCTGTTTGGCTTTTATACCACT 2088

** ** ** ******* * ** ************* *** * ***** ** ***** **

S288C_FLO1 CCAACAAACGTAACCCTAGAAATGACAGGTTATTTTTTACCACCACAGACGGGTTCTTAC 1456

RM11-1a_FLO1 CCAACAAACATCACCCTAGAAATGACAGGTTACTTTTTACCACCACAGACAGGTTCTTAC 2148

********* * ******************** ***************** *********

S288C_FLO1 ACATTCAAGTTTGCTACAGTTGACGACTCTGCAATTCTATCAGTAGGTGGTGCAACCGCG 1516

RM11-1a_FLO1 ACGTTTTCTTTTGCAACAATAGATGATTCTGCAATTTTATCAGTCGGTGGTAGCATTGCG 2208

** ** ***** *** * ** ** ********* ******* ****** * ***

S288C_FLO1 TTCAACTGTTGTGCTCAACAGCAACCGCCGATCACATCAACGAACTTTACCATTGACGGT 1576

RM11-1a_FLO1 TTCGAATGTTGTGCACAAGAACAACCTCCCATCACATCGACTAACTTCACCATCAATGGT 2268

*** * ******** *** * ***** ** ******** ** ***** ***** * ***

S288C_FLO1 ATCAAGCCATGGGGTGGAAGTTTGCCACCTAATATCGAAGGAACCGTCTATATGTACGCT 1636

RM11-1a_FLO1 ATCAAGCCATGGCATGGAAGTCTCCCTGATAATATCGCAGGGACTGTCTACATGTATGCT 2328

************ ******* * ** ******** *** ** ***** ***** ***

S288C_FLO1 GGCTACTATTATCCAATGAAGGTTGTTTACTCGAACGCTGTTTCTTGGGGTACACTTCCA 1696

RM11-1a_FLO1 GGTTTCTATTATCCAATGAAGATTGTTTACTCAAATGCCGTTTCCTGGGGTACACTTCCA 2388

** * **************** ********** ** ** ***** ***************

S288C_FLO1 ATTAGTGTGACACTTCCAGATGGTACCACTGTAAGTGATGACTTCGAAGGGTACGTCTAT 1756

RM11-1a_FLO1 ATTAGTGTGACACTACCAGATGGCACTACCGTTAGTGATGACTTTGAAGGGTACGTATAT 2448

************** ******** ** ** ** *********** *********** ***

S288C_FLO1 TCCTTTGACGATGACCTAAGTCAATCTAACTGTACTGTCCCTGACCCTTCAAATTATGCT 1816

RM11-1a_FLO1 ACCTTTGACAACAATCTAAGCCAGCCAAACTGTACCATTCCAGACCCTTCAAATTATACT 2508

******** * * ***** ** * ******** * ** *************** **

S288C_FLO1 GTCAGTACCACTACAACTACAACGGAACCATGGACCGGTACTTTCACTTCTACATCTACT 1876

RM11-1a_FLO1 GTCAGTACTACCATAACTACAACCGAGCCATGGACCGGTACTTTCACCTCTACGTCTACT 2568

******** ** * ********* ** ******************** ***** ******

S288C_FLO1 GAAATGACCACCGTCACCGGTACCAACGGCGTTCCAACTGACGAAACCGTCATTGTCATC 1936

RM11-1a_FLO1 GAGATGACTACTATCACTGGCACCAACGGTGTACCAACTGACGAAACCATCATTGTTGTC 2628

** ***** ** **** ** ******** ** *************** ******* **

S288C_FLO1 AGAACTCCAACAACTGCTAGCACCATCATAACTACAACTGAGCCATGGAACAGCACTTTT 1996

RM11-1a_FLO1 AAAACACCAACAACTGCTAGCACCATCATAACTACGACCGAAGCATGGA----------- 2677

* *** ***************************** ** ** ******

S288C_FLO1 ACCTCTACTTCTACCGAATTGACCACAGTCACTGGCACCAATGGTGTACGAACTGACGAA 2056

RM11-1a_FLO1 -------------------------------CTGGCAC---------------------- 2684

*******

S288C_FLO1 ACCATCATTGTAATCAGAACACCAACAACAGCCACTACTGCCATAACTACAACTGAGCCA 2116

RM11-1a_FLO1 ------------------------------------------------------------

S288C_FLO1 TGGAACAGCACTTTTACCTCTACTTCTACCGAATTGACCACAGTCACCGGTACCAATGGT 2176

RM11-1a_FLO1 ------------------------------------------------------------

S288C_FLO1 TTGCCAACTGATGAGACCATCATTGTCATCAGAACACCAACAACAGCCACTACTGCCATG 2236

RM11-1a_FLO1 ------------------------------------------------------------

S288C_FLO1 ACTACAACTCAGCCATGGAACGACACTTTTACCTCTACTTCTACCGAATTGACCACAGTC 2296

RM11-1a_FLO1 ------------------------------------------------------------

S288C_FLO1 ACCGGTACCAATGGTTTGCCAACTGATGAGACCATCATTGTCATCAGAACACCAACAACA 2356

RM11-1a_FLO1 ------------------------------------------------------------

S288C_FLO1 GCCACTACTGCCATGACTACAACTCAGCCATGGAACGACACTTTTACCTCTACTTCTACC 2416

RM11-1a_FLO1 -----------------------------------------TTTCACATCTACATCCACA 2703

*** ** ***** ** **

S288C_FLO1 GAATTGACCACAGTCACCGGTACCAATGGTTTGCCAACTGATGAGACCATCATTGTCATC 2476

RM11-1a_FLO1 GAAATGACCACCGTCACCGGTACCAACGGTTTGCCAACTGATGAAACCATTATCGTCATC 2763

*** ******* ************** ***************** ***** ** ******

S288C_FLO1 AGAACACCAACAACAGCCACTACTGCCATGACTACAACTCAGCCATGGAACGACACTTTT 2536

RM11-1a_FLO1 AGAACACCTACAACAGCAAGTACTGCCATAACTACAACTGAGCCATGGACTGGCACTTTT 2823

******** ******** * ********* ********* ********* * *******

S288C_FLO1 ACCTCTACATCCACTGAAATCACCACCGTCACCGGTACCAATGGTTTGCCAACTGATGAG 2596

RM11-1a_FLO1 ACATCTACACCCACAGAAATGACCACCGTCACCGGTACCAACGGTTTGCCAACTGATGAA 2883

** ****** **** ***** ******************** *****************

S288C_FLO1 ACCATCATTGTCATCAGAACACCAACAACAGCCACTACTGCCATGACTACACCTCAGCCA 2656

RM11-1a_FLO1 ACCATTATCGTCATCAGAACACCTACAACTGCCAACACCATCATAACTACGACCGAAGCA 2943

***** ** ************** ***** **** ** *** ***** * * **

S288C_FLO1 TGGAACGACACTTTTACCTCTACATCCACTGAAATGACCACCGTCACCGGTACCAACGGT 2716

RM11-1a_FLO1 TGGACTGGCACTTTCACCTCTACGTCTACTGAGATGACTACTATCACTGGCACCAACGGT 3003

**** * ****** ******** ** ***** ***** ** **** ** *********

S288C_FLO1 TTGCCAACTGATGAAACCATCATTGTCATCAGAACACCAACAACAGCCACTACTGCCATA 2776

RM11-1a_FLO1 GTACCAACTGACGAAACCATCATTGTTGTCAAAACACCAACAACTGATAGCACCATAATA 3063

* ******** ************** *** ************ * * ** ***

S288C_FLO1 ACTACAACTGAGCCATGGAACAGCACTTTTACCTCTACATCCACTGAAATGACCACCGTC 2836

RM11-1a_FLO1 ACTACAACCGAACCANNNNNNNNNNNNNNNNNNNNNNNNNNNNNNNNNNNNNNNNNNNNN 3123

******** ** ***

S288C_FLO1 ACCGGTACCAACGGTTTGCCAACTGATGAAACCATCATTGTCATCAGAACACCAACAACA 2896

RM11-1a_FLO1 NNNNNNNNNNNNNNNNNNNNNNNNNNNNNNNNNNNNNNNNNNNNNNNNNNNNNNNNNNNN 3183

S288C_FLO1 GCCACTACTGCCATAACTACAACTCAGCCATGGAACGACACTTTTACCTCTACATCCACT 2956

RM11-1a_FLO1 NNNNNNNNNNNNNNNNNNNNNNNNNNNNNNNNNNNNNNNNNNNNNNNNNNNNNNNNNNNN 3243

S288C_FLO1 GAAATGACCACCGTCACCGGTACCAACGGTTTGCCAACTGATGAAACCATCATTGTCATC 3016

RM11-1a_FLO1 NNNNNNNNNNNNNNNNNNNNNNNNNNNNNNNNNNNNNNNNNNNNNNNNNNNNNNNNNNNN 3303

S288C_FLO1 AGAACACCAACAACAGCCACTACTGCCATGACTACAACTCAGCCATGGAACGACACTTTT 3076

RM11-1a_FLO1 NNNNNNNNNNNNNNNNNNNNNNNNNNNNNNNNNNNNNNNNNNNNNNNNNNNNNNNNNNNN 3363

S288C_FLO1 ACCTCTACATCCACTGAAATCACCACCGTCACCGGTACCACCGGTTTGCCAACTGATGAG 3136

RM11-1a_FLO1 NNNNNNNNNNNNNNNNNNNNNNNNNNNNNNNNNNNNNNNNNNNNNNNNNNNNNNNNNNNN 3423

S288C_FLO1 ACCATCATTGTCATCAGAACACCAACAACAGCCACTACTGCCATGACTACAACTCAGCCA 3196

RM11-1a_FLO1 NNNNNNNNNNNNNNNNNNNNNNNNNNNNNNNNNNNNNNNNNNNNNNNNNNNNNNNNNNNN 3483

S288C_FLO1 TGGAACGACACTTTTACCTCTACATCCACTGAAATGACCACCGTCACCGGTACCAACGGC 3256

RM11-1a_FLO1 NNNNNNNNNNNNNNNNNNNNNNNNNNNNNNNNNNNNNNNNNNNNNNNNNNNNNNNNNNNN 3543

S288C_FLO1 GTTCCAACTGACGAAACCGTCATTGTCATCAGAACTCCAACTAGTGAAGGTCTAATCAGC 3316

RM11-1a_FLO1 NNNNNNNNNNNNNNNNNNNNNNNNNNNNNNNNNNNNNNNNNNNNNNNNNNNNNNNNNNNN 3603

S288C_FLO1 ACCACCACTGAACCATGGACTGGTACTTTCACCTCTACATCCACTGAGATGACCACCGTC 3376

RM11-1a_FLO1 NNNNNNNNNNNNNNNNNNNNNNNNNNNNNNNNNNNNNNNNNNNNNNNNNNNNNNNNNNNN 3663

S288C_FLO1 ACCGGTACTAACGGTCAACCAACTGACGAAACCGTGATTGTTATCAGAACTCCAACCAGT 3436

RM11-1a_FLO1 NNNNNNNNNNNNNNNNNNNNNNNNNNNNNNNNNNNNNNNNNNNNNNNNNNNNNNNNNNNN 3723

S288C_FLO1 GAAGGTTTGGTTACAACCACCACTGAACCATGGACTGGTACTTTTACTTCTACATCTACT 3496

RM11-1a_FLO1 NNNNNNNNNNNNNNNNNNNNNNNNNNNNNNNNNNNNNNNNNNNNNNNNNNNNNNNNNNNN 3783

S288C_FLO1 GAAATGACCACCATTACTGGAACCAACGGCGTTCCAACTGACGAAACCGTCATTGTCATC 3556

RM11-1a_FLO1 NNNNNNNNNNNNNNNNNNNNNNNNNNNNNNNNNNNNNNNNNNNNNNNNNNNNNNNNNNNN 3843

S288C_FLO1 AGAACTCCAACCAGTGAAGGTCTAATCAGCACCACCACTGAACCATGGACTGGTACTTTT 3616

RM11-1a_FLO1 NNNNNNNNNNNNNNNNNNNNNNNNNNNNNNNNNNNNNNNNNNNNNNNNNNNNNNNNNNNN 3903

S288C_FLO1 ACTTCTACATCTACTGAAATGACCACCATTACTGGAACCAATGGTCAACCAACTGACGAA 3676

RM11-1a_FLO1 NNNNNNNNNNNNNNNN-----------------------------------NNTGAAGGA 3928

*** * *

S288C_FLO1 ACCGTTATTGTTATCAGAACTCCAACTAGTGAAGGTCTAATCAGCACTACAACGGAACCA 3736

RM11-1a_FLO1 A-----------ATTTAGACTATAAATAAAAAAA--------AAAAAAAAAAGAAAAATA 3969

* ** *** ** ** ** * * * ** ** *

S288C_FLO1 TGGACCGGTACTTTCACTTCTACATCTACTGAAATGACGCACGTCACCGGTACCAACGGC 3796

RM11-1a_FLO1 AAAATAAAAACATTCAC--------------AAATG------------------------ 3991

* ** ***** *****

S288C_FLO1 GTTCCAACTGACGAAACCGTCATTGTCATCAGAACTCCAACCAGTGAAGGTCTAATCAGC 3856

RM11-1a_FLO1 -------------------------------GAATACCAAGTA--TAAAGTCTA------ 4012

*** **** * ** *****

S288C_FLO1 ACCACCACTGAACCATGGACTGGCACTTTCACTTCGACTTCCACTGAGGTTACCACCATC 3916

RM11-1a_FLO1 --------------------------------------------------TCTCATCAT- 4021

* ** ***

S288C_FLO1 ACTGGAACCAACGGTCAACCAACTGACGAAACTGTGATTGTTATCAGAACTCCAACCAGT 3976

RM11-1a_FLO1 ------ACCCAATGTTATCCGATTGG---------------------------ATCCAGG 4048

*** * ** * ** * ** * ****

S288C_FLO1 GAAGGTCTAATCAGCACCACCACTGAACCATGGACTGGTACTTTCACTTCTACATCTACT 4036

RM11-1a_FLO1 CCGGGCCCAAT-------------------------------TTTACTT----------- 4066

** * *** ** ****

S288C_FLO1 GAAATGACCACCGTCACCGGTACTAACGGTCAACCAACTGACGAAACCGTGATTGTTATC 4096

RM11-1a_FLO1 ------------ACCCCCTGTACTCATTGGCAAACAAT---------------------- 4092

* ** ***** * * *** ***

S288C_FLO1 AGAACTCCAACCAGTGAAGGTTTGGTTACAACCACCACTGAACCATGGACTGGTACTTTT 4156

RM11-1a_FLO1 -------------------------------------------CATAAACAGATTTTTTA 4109

*** ** * * ***

S288C_FLO1 ACTTCGACTTCCACTGAAATGTCTACTGTCACTGGAACCAATGGCTTGCCAACTGATGAA 4216

RM11-1a_FLO1 ATTCCCACCT----------------TATCCATAGGGACAACAGC--------------- 4138

* * * ** * * ** * * *** **

S288C_FLO1 ACTGTCATTGTTGTCAAAACTCCAACTACTGCCATCTCATCCAGTTTGTCATCATCATCT 4276

RM11-1a_FLO1 ---------------------CCAGGAACTGC------------TTTTTCATTACCACTT 4165

*** ***** *** **** * ** *

S288C_FLO1 TCAGGACAAATCACCAGCTCTATCACGTCTTCGCGTCCAATTATTACCCCATTCTATCCT 4336

RM11-1a_FLO1 ---------GTTACCAGTG----------------------------------------T 4176

* ***** *

S288C_FLO1 AGCAATGGAACTTCTGTGATTTCTTCCTCAGTAATTTCTTCCTCAGTCACTTCTTCTCTA 4396

RM11-1a_FLO1 AACAACAAG------------------TCGGAAAT----------------------CTA 4196

* *** ** * *** ***

S288C_FLO1 TTCACTTCTTCTCCAGTCATTTCTTCCTCAGTCATTTCTTCTTCTACAACAACCTCCACT 4456

RM11-1a_FLO1 TTCA------------TCATTACCACCT-----GTTTCCAGTGCGACAACAA-------- 4231

**** ***** * *** **** * * *******

S288C_FLO1 TCTATATTTTCTGAATCATCTAAATCATCCGTCATTCCAACCAGTAGTTCCACCTCTGGT 4516

RM11-1a_FLO1 ---------------------------------------GCCAGGA-------------- 4238

**** *

S288C_FLO1 TCTTCTGAGAGCGAAACGAGTTCAGCTGGTTCTGTCTCTTCTTCCTCTTTTATCTCTTCT 4576

RM11-1a_FLO1 -----------------------AACTGATTTT--------------------------- 4248

* *** ** *

S288C_FLO1 GAATCATCAAAATCTCCTACATATTCTTCTTCATCATTACCACTTGTTACCAGTGCGACA 4636

RM11-1a_FLO1 ---------------------------------TCATACCCACCTGTTACCAGTGCGACA 4275

**** **** ****************

S288C_FLO1 ACAAGCCAGGAAACTGCTTCTTCATTACCACCTGCTACCACTACAAAAACGAGCGAACAA 4696

RM11-1a_FLO1 ACAAGCCAGGATACTGCTTATTCATTACCACCTGCTACCA-TACAAAAACGAGCGAACAA 4334

*********** ******* ******************** *******************

S288C_FLO1 ACCACTTTGGTTACCGTGACATCCTGCGAGTCTCATGTGTGCACTGAATCCATCTCCCCT 4756

RM11-1a_FLO1 ACCACTTTGGTTACCGTGACATCCTGCGAATCTCATGTGTGCA-TGAATCCATCTCCTCT 4393

***************************** ************* ************* **

S288C_FLO1 GCGATTGTTTCCACAGCTACTGTTACTGTTAGCGGCGTCACAA-CAGAG-TATACCACAT 4814

RM11-1a_FLO1 GCGATTGTTTCCACGGCCACCGTTATTGTTAGCGGTGTCACAAACAGAGATATACCACAT 4453

************** ** ** **** ********* ******* ***** **********

S288C_FLO1 GGTGCCCTATTTCTACTACAGAGA---------------------CAACAAAGCAAACCA 4853

RM11-1a_FLO1 GGTGCCCTATTTCTACCACAGAGATAACAAAGCAAACTACGGAGACAACAAAGCAAACCA 4513

**************** ******* ***************

S288C_FLO1 AAGGGACAACAGAGCAAACCACAGAAACAACAAAACAAACCACGGTAGTTACAATTTCTT 4913

RM11-1a_FLO1 AGGGGACAACAGAGCAAACCACAGAAACAACAAAACAAACCACAGTAGTTACAATTTCTT 4573

* ***************************************** ****************

S288C_FLO1 CTTGTGAATCTGACGTATGCTCTAAGACTGCTTCTCCAGCCATTGTATCTACAAGCACTG 4973

RM11-1a_FLO1 CTTGTGAATCTGACATATGCTCTAAGACTGCTTCTCCAGCCATTGTGTCTACAAGCACTG 4633

************** ******************************* *************

S288C_FLO1 CTACTATTAACGGCGTTACTACAGAATACACAACATGGTGTCCTATTTCCACCACAGAAT 5033

RM11-1a_FLO1 CTACTATTAACGGCGTTACCACAGAATACACAACATGGTGTCCTATTTCCACCACAGAAT 4693

******************* ****************************************

S288C_FLO1 CGAGGCAACAAACAACGCTAGTTACTGTTACTTCCTGCGAATCTGGTGTGTGTTCCGAA- 5092

RM11-1a_FLO1 CGAAGCAACAAACTACGCTAGTTACTGTTACTTCCTGCGAATCTGGTGTGTGTTCCGAAT 4753

*** ********* *********************************************

S288C_FLO1 -ACTGCTTCACCTGCCATTGTTTCGACGGCCACGGCTACTGTGAATGATGTTGTTACGGT 5151

RM11-1a_FLO1 CACTGCTTCACCTGCCATTGTTTCGACGGCCACGGCTACTGTGAATGATGTTGTTACGGT 4813

***********************************************************

S288C_FLO1 CTATCCTACATGGAGGCCACAGACTGCGAATGAAGAGTCTGTCAGCTCTAAAATGAACAG 5211

RM11-1a_FLO1 CTATCCTACATGGAGGCCACAGACTACGAATGAACAGTCTGTCAGCTCTAAAATGAACAG 4873

************************* ******** *************************

S288C_FLO1 TGCTACCGGTGAGACAACAACCAATACTTTAGCTGCTGAAACGACTACCAATACTGTAGC 5271

RM11-1a_FLO1 TGCTACCAGTGAGACAAC------------------------------------------ 4891

******* **********

S288C_FLO1 TGCTGAGACGATTACCAATACTGGAGCTGCTGAGACGAAAACAGTAGTCACCTCTTCGCT 5331

RM11-1a_FLO1 ------------TACCAATACTGGGGCTGCTGAGACAAAAACAGCAGTCACCTCTTCACT 4939

************ *********** ******* ************ **

S288C_FLO1 TTCAAGATCTAATCACGCTGAAACACAGACGGCTTCCGCGACCGATGTGATTGGTCACAG 5391

RM11-1a_FLO1 TTCAAGATTCAATCACGCTGAAACACAGACGGCTTCCGCGACCGATGTGATTGGTCACAG 4999

******** **************************************************

S288C_FLO1 CAGTAGTGTTGTTTCTGTATCCGAAACTGGCAACACCAAGAGTCTAACAAGTTCCGGGTT 5451

RM11-1a_FLO1 CAGTAGTGTTGTTTCTGTATCCGAAACTGGCAACACCAAGAGTCTAACAAGTTCCGGGTT 5059

************************************************************

S288C_FLO1 GAGTACTATGTCGCAACAGCCTCGTAGCACACCAGCAAGCAGCATGGTAGGATATAGTAC 5511

RM11-1a_FLO1 GAGCACTATGTCGCAACAGCCTCGTAGCACACCAGCAAGTAGCATGGTAGGATCTAGTAC 5119

*** *********************************** ************* ******

S288C_FLO1 AGCTTCTTTAGAAATTTCAACGTATGCTGGCAGTGCCAACAGCTTACTGGCCGGTAGTGG 5571

RM11-1a_FLO1 AGCTTCTTTAGAAATTTCAACGTATGCTGGCAGTGCCAACAGCTTACTGGCCGGTAGTGG 5179

************************************************************

S288C_FLO1 TTTAAGTGTCTTCATTGCGTCCTTATTGCTGGCAATTATTTAATAAAATTCGCGTTCTTT 5631

RM11-1a_FLO1 TTTAAGTGTCTTCATTGCGTCCTTATTGCTGGCAATTATTTAATAAAATTCGCGTTCTTT 5239

************************************************************

S288C_FLO1 TTACGTATCTGTGTATCTTTTCTTTGCTAATTATACGCTGACATGAATTATTTTTTAACT 5691

RM11-1a_FLO1 TTACGTATCTGTGTATCTTTTCTTTGCTAAATATACGCTGGCATGAATTATTTTTTAACT 5299

****************************** ********* *******************

S288C_FLO1 GTTTCTCCTCCATACTTTCAAATATTCAAATTGA-CTAAATGATAATTCTTGCGCTTCTT 5750

RM11-1a_FLO1 GTTTCTTCTCCATACTTTCAAATATTCAAATTGAACTAAATGATAATTCTTGCGCTTCCT 5359

****** *************************** *********************** *

S288C_FLO1 ATTTTGAAAAAGTAGATATGTGTATCATAAAGAAAACGTTATTATTATTGTCTTAGGCAA 5810

RM11-1a_FLO1 ATTTTGAAAAAGTAGATATGTGTATCATAAAGAAAACGTTACTATTATTGTCTTAGGCAA 5419

***************************************** ******************

S288C_FLO1 CAAAAATCCATGAAAAGAATTTTACCGTTATCGATATCATTGTATTTATTTTATTTATTT 5870

RM11-1a_FLO1 CAAAAATCCATGAAAAGAATTTTACCGTTATCGATACCATTGTATTTATTTTATTTATTT 5479

************************************ ***********************

S288C_FLO1 ATTCAATTTTTTTTTTTTT--GGTTTATATCCTGCAAACAACACTTCGAATTCAATTCGA 5928

RM11-1a_FLO1 ATTTAATTTTTTTTTTTTTTTGGTTTATATCCTGCAAACAACACTTCGAATTCAATTCGA 5539

*** *************** ***************************************

S288C_FLO1 TATTTCATAAGTTACAACTAACACTTATAGAAACCGATGTATGAGTACTTATTATTAACG 5988

RM11-1a_FLO1 TATTTCATAAGTTACAACTAACACTTATAGAAACCGATGTATGAGTACTTATTATTAACG 5599

************************************************************

S288C_FLO1 AGGAAAAATGCCCTATTTTCTTTAGCAATTAATGAACCATCGCCAACTTTTGCTTTAACA 6048

RM11-1a_FLO1 AGGAAAAATGGCCTATTTTGTTTAGCAATCAATGAACCATCGCCAATTTTTGCTTTAACA 5659

********** ******** ********* **************** *************

S288C_FLO1 ATTATTGCCATTTTCAGCAGTACTAACGTAAGATCTAGTGTGGTTCGCTTAGGATGTTTT 6108

RM11-1a_FLO1 ATTATTGCCATTTTCAGTAGTACTAACGTAAGATCTAGTGTGGTTCGCTTAGGATGTTTT 5719

***************** ******************************************

S288C_FLO1 CGAGTAGAAATCTGCTGCACATGCCACACGCAGTACTTGAAACTTGAAATAATGGTGATA 6168

RM11-1a_FLO1 TGAGTAGAAATCTGCT-TACATGCCACACGCAGTACTTGAAACTTGAAATAATGGGGATA 5778

*************** ************************************* ****

S288C_FLO1 ATTAGTTATTTAAAGTATGTTAATCTTCCTTGTTCTTTTATATTTATTTCGAATTCTTTT 6228

RM11-1a_FLO1 ATTAGTTATTTAAAGTATGTG-------CTTGTTCTTTTATATTTATTTCGAATTCTTTT 5831

******************** ********************************

S288C_FLO1 GCACTAGTATTTAAAATATCAGCAGAGGTGTAAAAGTGCACCAAAATTATTGTAAAACTA 6288

RM11-1a_FLO1 GCACTAGTATTTAAAATATCAGCAGAGGCGTAAAAGTGCACCAAAATTATTGTAAAACTA 5891

**************************** *******************************

S288C_FLO1 CTTGCCCTAAAATTGATACTTCATACTTGACATATTCAAAAGGGGTCCAAGTATAGATGC 6348

RM11-1a_FLO1 CTTGCCCTAAAATTGATACTTCATACTTGACATATTCAAAAGGGGTCCAAGTATAGATGC 5951

************************************************************

S288C_FLO1 ATCAAAAAAAAAAA--TTATCCGATGATGAGCAAATGGTAGCTTTTCGTTCCC-AGGAAG 6405

RM11-1a_FLO1 ATCAAAAAAAAAAAAATTATCCGATGATGAGCAAATGGTAGCTTTTCGTTCCCCAGGAAG 6011

************** ************************************* ******

S288C_FLO1 TGTAGTAGTTCCATGAAGTCTAATGAGACTTTGGAAAAAGGTTTGTCACGAGCACCTAAC 6465

RM11-1a_FLO1 TGTAGTAGTTCCATGAAGTCTAATGAGACTTTGGAAAAAGGTTTGTCACGAGCACCGAAC 6071

******************************************************** ***

S288C_FLO1 TATTGTATTTTGGAATTTTGATAAACTTCAAAACGGGAACGAAGTGTTAAACTTAGATGC 6525

RM11-1a_FLO1 TACTGTATTTTGGAATTTTGATAAACTTCAAAACGGGAACGAAGTGTTAAACTTAGATGC 6131

** *********************************************************

S288C_FLO1 GGTTGATTTAAGCTTTAAAAGAGGAAAATAATGACTGATGATAAGAAGTCAACAACGATT 6585

RM11-1a_FLO1 GGTTGTTTTAAGCTTTAAAAGAGGAAAATAATGACTGGTGATAAGAAGTCAACAACGATT 6191

***** ******************************* **********************

S288C_FLO1 CAAAGCAGGTGAATTTCCATTACGTTTCG 6614

RM11-1a_FLO1 CAAAGCAGGTGAATTTCCATTACGTTTCG 6220

*****************************

RM11-1a_SWH1 GTTTATGCTGAGTTTTTGCGCATCAATATTATTTTTACTACTACTAATACTACTACTAC- 59

S288c_SWH1 GTTTATGCTGAGTTTTTGCGCATCAATATTATTTTTACTACTACTACTACTACTACTACT 60

********************************************** ************

RM11-1a_SWH1 -----ATACTATTAAATATACTAAAAATAAGAGGAAAACGCTTTGGAAGTGACTGGCGCC 114

S288c_SWH1 ACTACATACTATTAAATATACTAA--ATAAGAGGAAAACGCTTTGGAAGTGACTGGCGCC 118

******************* **********************************

RM11-1a_SWH1 GCCGCTGGCTACTATAATAGCAGCGACTGTAATTTAATCTCATCCCGTCGTTCGCATTAC 174

S288C_SWH1 GCCGCTGGCTACTATAATAGCAGCGACTGTAATTTAATCTCATCCCGTCGTTTGGATTAC 178

**************************************************** * *****

RM11-1a_SWH1 CTCTTTTACTCGCCGAGCGAACGTGCACCAAAAAAGGAAAGGAAAAAAAGAAAAAAAAAG 234

S288C_SWH1 CTCTTTTACTCGCCGAGCGAACGTGCACCAAAAAGGGAAAGGAAAAAAAGAAAAAAAAAG 238

********************************** *************************

RM11-1a_SWH1 GAAAAAGGAAACTCAAAACTTGGATAAATAGAAGCATTCAAACTAAATTAAACTGCAAAA 294

S288C_SWH1 GAAAAAGGAAACTCAAAACTTGGATAAATAGAAGCACTCAAACTAAATTAAACTGCCAAA 298

************************************ ******************* ***

RM11-1a_SWH1 AAAAAAAAAAAAAAAATAAAAAGGGAAAAGTTTAAACATCAAAGTACACCTTTCACCCCT 354

S288C_SWH1 AAAAAAAA-----AAATAAAAAGGGAAAAGTTTAAACATCAAAGTACACCTTTCACCCCT 353

******** ***********************************************

RM11-1a_SWH1 CCACACACTATGGAACAACCTGATCTATCGTCTGTGGCCATCAGTAAGCCGCTGCTGAAG 414

S288C_SWH1 CCACACACCATGGAACAACCTGATCTATCGTCTGTGGCCATCAGTAAGCCGCTGCTGAAG 413

******** ***************************************************

RM11-1a_SWH1 TTGAAACTTCTCGACGCCCTTCGTCAGGGAAGTTTCCCCAACCTACAAGATCTCCTAAAG 474

S288C_SWH1 TTGAAACTTCTCGACGCCCTTCGCCAGGGAAGTTTCCCCAACCTACAAGATCTCCTAAAG 473

*********************** ************************************

RM11-1a_SWH1 AAACAATTCCAGCCGCTAGACGACCCAAACGTCCAACAAGTGCTCCATCTCATGCTCCAC 534

S288C_SWH1 AAACAATTCCAGCCGCTAGACGACCCAAACGTCCAACAAGTGCTCCATCTCATGCTCCAC 533

************************************************************

RM11-1a_SWH1 TATGCTGTGCAAGTCGCCCCCATGGCTGTCATAAAGGAAATCGTCCATCATTGGGTCTCA 594

S288C_SWH1 TATGCCGTGCAAGTCGCCCCCATGGCTGTCATAAAGGAAATCGTCCATCATTGGGTCTCA 593

***** ******************************************************

RM11-1a_SWH1 ACTACAAACACCACTTTTCTAAACATCCATCTTGATCTAAACGAACGGGACTCCAACGGC 654

S288C_SWH1 ACTACAAACACCACTTTTCTAAACATCCATCTTGATCTAAACGAACGGGACTCCAACGGC 653

************************************************************

RM11-1a_SWH1 AACACCCCATTGCACATCGCCGCCTACCAGTCCCGCGGTGATATAGTAGCCTTCCTCCTG 714

S288C_SWH1 AACACCCCATTGCACATCGCCGCCTACCAGTCCCGCGGTGATATCGTAGCCTTCCTCCTG 713

******************************************** ***************

RM11-1a_SWH1 GACCAACCAACCATCAACGACTGCGTGCTCAACAACTCCCACTTGCAGGCCATCGAAATG 774

S288C_SWH1 GACCAACCAACCATCAACGACTGCGTGCTCAACAACTCCCACTTGCAGGCCATCGAAATG 773

************************************************************

RM11-1a_SWH1 TGCAAGAACCTAAACATCGCGCAGATGATGCAGGTGAAACGCTCCACATACGTTGCAGAG 834

S288C_SWH1 TGCAAGAACCTAAACATCGCGCAGATGATGCAGGTGAAACGCTCCACATACGTTGCAGAG 833

************************************************************

RM11-1a_SWH1 ACCGCCCAGGAATTCAGAACAGCTTTTAACAACAGGGACTTCGGCCACCTAGAATCTATC 894

S288C_SWH1 ACCGCCCAGGAATTCAGAACAGCTTTTAACAACAGGGACTTCGGCCACCTAGAATCTATC 893

************************************************************

RM11-1a_SWH1 CTCTCCAGCCCTCGAAACGCAGAACTGCTCGACATCAACGGTATGGACCCGGAGACTGGC 954

S288C_SWH1 CTCTCCAGCCCTCGAAACGCAGAACTGCTCGACATCAACGGTATGGACCCGGAGACTGGC 953

************************************************************

RM11-1a_SWH1 GATACCGTTCTGCACGAATTCGTCAAGAAAAGAGACGTCATCATGTGCCGCTGGTTGCTT 1014

S288C_SWH1 GATACCGTTCTGCACGAATTCGTCAAGAAAAGAGACGTCATCATGTGCCGTTGGTTGCTT 1013

************************************************** *********

RM11-1a_SWH1 GAACACGGTGCTGACCCCTTCAAGAGAGACCGCAAGGGCAAACTGCCCATCGAGCTCGTT 1074

S288C_SWH1 GAACACGGTGCTGACCCCTTCAAGAGAGACCGCAAGGGCAAACTGCCCATCGAGCTCGTT 1073

************************************************************

RM11-1a_SWH1 AGGAAAGTCAATGAAAACGACACCGCCACCAACACCAAGATCGCCATCGACATCGAACTG 1134

S288C_SWH1 AGGAAAGTCAATGAAAACGACACCGCCACCAACACCAAGATCGCCATCGACATCGAACTG 1133

************************************************************

RM11-1a_SWH1 AAAAAACTATTGGAAAGGGCCACCAGGGAGCAAAGTGTCATCGACGTCACAAACAACAAC 1194

S288C_SWH1 AAAAAACTATTGGAAAGGGCCACCAGGGAGCAAAGTGTCATCGACGTCACAAACAACAAC 1193

************************************************************

RM11-1a_SWH1 TTACACGAGGCCCCCACTTACAAAGGCTACCTGAAAAAATGGACCAACTTTGCTCAAGGC 1254

S288C_SWH1 TTGCACGAGGCCCCCACTTACAAAGGCTACCTGAAAAAATGGACCAACTTCGCTCAAGGC 1253

** *********************************************** *********

RM11-1a_SWH1 TACAAATTGCGTTGGTTCATCCTTAGTAGCGATGGGAAACTATCCTACTACATCGATCAG 1314

S288C_SWH1 TACAAATTGCGTTGGTTCATCCTTAGTAGCGATGGGAAACTATCCTACTACATCGATCAG 1313

************************************************************

RM11-1a_SWH1 GCCGACACTAAGAATGCCTGCAGGGGCTCCCTAAACATGTCTTCGTGCTCTCTGCATTTG 1374

S288C_SWH1 GCCGACACTAAGAATGCCTGCAGGGGCTCCCTAAACATGTCTTCGTGCTCTCTGCATTTG 1373

************************************************************

RM11-1a_SWH1 GATTCGTCTGAAAAGTTGAAATTCGAAATTATCGGCGGTAACAACGGTGTTATCAGGTGG 1434

S288C_SWH1 GATTCGTCTGAAAAGTTGAAATTCGAAATTATCGGCGGTAACAACGGTGTTATCAGGTGG 1433

************************************************************

RM11-1a_SWH1 CATTTAAAGGGGAACCACCCCATCGAGACAAATAGATGGGTTTGGGCCATCCAGGGCGCC 1494

S288C_SWH1 CATTTAAAGGGGAACCACCCCATCGAGACAAATAGATGGGTTTGGGCCATCCAGGGCGCC 1493

************************************************************

RM11-1a_SWH1 ATAAGATACGCAAAGGACAGAGAAATTTTGCTGCACAATGGCCCCTATTCGCCTTCTCTG 1554

S288C_SWH1 ATAAGATACGCAAAGGACAGAGAAATTTTGCTGCACAATGGCCCCTATTCGCCTTCTCTG 1553

************************************************************

RM11-1a_SWH1 GCCTTAAGCCATGGCTTGTCATCCAAAGTGTCCAATAAAGAAAACTTGCATGCAACTTCA 1614

S288C_SWH1 GCCTTAAGCCATGGCTTGTCATCCAAAGTGTCCAATAAAGAAAACTTGCATGCAACTTCA 1613

************************************************************

RM11-1a_SWH1 AAACGGTTGACCAAGAGCCCGCATCTGTCCAAATCCACACTGACACAAAACGATCACGAT 1674

S288C_SWH1 AAACGGTTGACCAAGAGCCCGCATCTGTCCAAATCCACACTGACACAAAACGATCACGAT 1673

************************************************************

RM11-1a_SWH1 AATGACGATGACAGCACTAACAACAACAACAACAAAAGTAATAATGATTATGACGATAAT 1734

S288C_SWH1 AATGACGATGACAGCACTAACAACAACAACAACAAAAGTAATAATGATTATGACGATAAT 1733

************************************************************

RM11-1a_SWH1 AATAATAATAATAATAATAATAATAATGACGATGATGATTATGATGATGATGAAAGTAGA 1794

S288C_SWH1 AATAATAATAATAATAATG------ACGATGATGATTATGATGATGATGATGAAAGTAGA 1787

****************** * ** ****** ** ********************

RM11-1a_SWH1 CCCCTCATAGAACCATTACCGTTGATTTCATCCAGAAGCCAAAGCTTAAGCGAAATCGCT 1854

S288C_SWH1 CCCCTCATAGAACCATTACCGTTGATTTCATCCAGAAGCCAAAGCTTAAGCGAAATCACT 1847

********************************************************* **

RM11-1a_SWH1 TCCGGTCCACATTCTAGGAAGTCTACAGTCTCGTCTACAAGGGCAGCCGATATACCATCA 1914

S288C_SWH1 CCCGGTCCACATTCTAGGAAGTCTACAGTCTCGTCTACAAGGGCAGCCGATATACCATCA 1907

***********************************************************

RM11-1a_SWH1 GACGACGAGGGTTACTCTGAGGACGATTCTGATGACGACGGTAACTCCTCTTACACAATG 1974

S288C_SWH1 GATGATGAGGGTTACTCTGAGGACGATTCTGATGACGACGGTAACTCCTCTTACACAATG 1967

** ** ******************************************************

RM11-1a_SWH1 GAAAACGGCGGTGAAAATGATGGCGACGAAGATCTAAATGCCATTTATGGTCCCTATATT 2034

S288C_SWH1 GAAAACGGCGGTGAAAACGATGGCGACGAAGATCTAAATGCCATTTATGGTCCCTATATT 2027

***************** ******************************************

RM11-1a_SWH1 CAAAAAATACACATGCTACAAAGATCCATTTCCATCGAGTTGGCATCTTTGAACGAATTG 2094

S288C_SWH1 CAAAAACTACACATGCTACAAAGATCCATTTCCATCGAGTTGGCATCTTTGAACGAATTG 2087

****** *****************************************************

RM11-1a_SWH1 CTGCAAGATAAACAACAACACGATGAGTACTGGAACACCGTCAACACTTCTATTGAAACC 2154

S288C_SWH1 CTGCAAGATAAACAACAACACGATGAGTACTGGAACACCGTCAACACTTCTATTGAAACC 2147

************************************************************

RM11-1a_SWH1 GTCAGCGAATTTTTCGACAAATTAAATCGGTTGACCTCTCAAAGGGAAAAAAGAATGATT 2214

S288C_SWH1 GTCAGCGAATTTTTCGACAAATTAAATCGGTTGACCTCTCAAAGGGAAAAAAGAATGATT 2207

************************************************************

RM11-1a_SWH1 GCCCAAATGACTAAGCAACGGGATGTTAACAATGTATGGATTCAATCGGTAAAAGATCTG 2274

S288C_SWH1 GCCCAAATGACCAAGCAACGGGATGTTAACAATGTTTGGATTCAATCGGTAAAAGATCTG 2267

*********** *********************** ************************

RM11-1a_SWH1 GAAATGGAACTGGTTGATAAAGACGAAAAATTGGTTGCCTTGGATAAAGAACGGAAAAAT 2334

S288C_SWH1 GAAATGGAACTGGTTGATAAAGACGAAAAATTGGTTGCCTTGGATAAAGAACGGAAAAAT 2327

************************************************************

RM11-1a_SWH1 CTGAAAAAAATGCTTCAAAAAAAATTGAACAATCAACCACAGATTGAAACTGAGGCTAAT 2394

S288C_SWH1 CTGAAAAAAATGCTTCAAAAAAAATTGAACAATCAACCACAGGTTGAAACTGAGGCTAAT 2387

****************************************** *****************

RM11-1a_SWH1 GAAGAATCCGATGATGCAAATTCAATGATAAAAGGATCCCAAGAATCAACAAATACTCTT 2454

S288C_SWH1 GAAGAATCCGATGATGCAAATTCAATGATAAAAGGATCCCAAGAATCAACAAATACCCTT 2447

******************************************************** ***

RM11-1a_SWH1 GAGGAAATCGTAAAATTTATCGAAGCAACAAAGGAAAGTGATGAGGATTCTGACGCCGAC 2514

S288C_SWH1 GAGGAAATCGTAAAATTTATCGAAGCAACAAAGGAAAGTGATGAGGATTCTGACGCCGAC 2507

************************************************************

RM11-1a_SWH1 GAATTTTTCGACGCAGAAGAAGCTGCTTCCGACAAAAAAGCCAATGATTCGGAAGACTTA 2574

S288C_SWH1 GAATTTTTCGACGCAGAAGAAGCTGCTTCCGACAAAAAAGCCAATGATTCGGAAGACTTA 2567

************************************************************

RM11-1a_SWH1 ACCACAAACAAGGAGACTCCAGCTAATGCGAAACCACAAGAAGTAGCTCCTGAAGACGAG 2634

S288C_SWH1 ACCACAAACAAGGAGACTCCAGCTAATGCGAAACCACAAGAAGAAGCTCCTGAAGACGAG 2627

******************************************* ****************

RM11-1a_SWH1 AGCCTTATTGTGATCAGTTCTCCACAGGTGGAAAAGAAGAACCAACTATTAAAAGAGGGA 2694

S288C_SWH1 AGCCTTATTGTGATCAGTTCTCCACAGGTGGAAAAGAAGAACCAACTATTAAAAGAGGGA 2687

************************************************************

RM11-1a_SWH1 TCATTCGTCGGATATGAAGACCCAGTGAGAACCAAACTGGCTTTAGACGAAGATAATCGT 2754

S288C_SWH1 TCATTCGTCGGATATGAAGACCCAGTGAGAACCAAACTGGCTTTAGACGAAGATAATCGT 2747

************************************************************

RM11-1a_SWH1 CCCAAGATTGGTCTCTGGTCTGTTTTAAAGTCTATGGTCGGTCAAGACTTAACCAAACTA 2814

S288C_SWH1 CCCAAGATTGGTCTCTGGTCTGTTTTAAAGTCTATGGTCGGTCAAGACTTAACCAAACTA 2807

************************************************************

RM11-1a_SWH1 ACTCTACCGGTATCGTTCAATGAGCCAACATCCTTACTACAGAGAGTATCTGAAGATATT 2874

S288C_SWH1 ACTCTACCGGTATCGTTCAATGAGCCAACATCCTTACTACAGAGAGTATCTGAAGATATT 2867

************************************************************

RM11-1a_SWH1 GAGTATTCTCATATTCTTGACCAAGCTGCCACTTTTGAAGACTCCTCTTTAAGAATGCTA 2934

S288C_SWH1 GAGTATTCTCATATTCTTGACCAAGCTGCCACTTTTGAAGACTCCTCTTTAAGAATGCTA 2927

************************************************************

RM11-1a_SWH1 TATGTAGCTGCCTTTACTGCATCAATGTACGCATCTACCACTAACAGAGTGTCTAAACCA 2994

S288C_SWH1 TATGTAGCTGCCTTTACTGCATCAATGTACGCATCTACCACTAACAGAGTGTCTAAACCA 2987

************************************************************

RM11-1a_SWH1 TTCAACCCCTTACTCGGTGAAACTTTTGAATATGCCAGAACTGATGGTCAGTACCGATTC 3054

S288C_SWH1 TTCAACCCCTTACTCGGTGAAACTTTTGAATATGCCAGAACTGATGGTCAGTACCGATTC 3047

************************************************************

RM11-1a_SWH1 TTCACCGAACAAGTCTCTCACCACCCACCTATCTCTGCTACTTGGACAGAATCGCCCAAA 3114

S288C_SWH1 TTCACCGAACAAGTCTCTCACCACCCACCTATCTCTGCTACTTGGACAGAATCGCCCAAA 3107

************************************************************

RM11-1a_SWH1 TGGGATTTTTACGGTGAATGTAATGTTGATTCGTCATTCAATGGGCGCACGTTCGCCGTG 3174

S288C_SWH1 TGGGATTTTTACGGTGAATGTAATGTTGATTCGTCATTCAATGGGCGCACGTTCGCCGTG 3167

************************************************************

RM11-1a_SWH1 CAACATTTAGGATTATGGTACATTACTATCCGGCCCGATCATAATATTAGTGTTCCCGAG 3234

S288C_SWH1 CAACATTTAGGATTATGGTACATTACTATCCGGCCTGATCATAATATTAGTGTTCCCGAG 3227

*********************************** ************************

RM11-1a_SWH1 GAAACTTATTCCTGGAAAAAACCAAATAACACTGTTATCGGTATTTTAATGGGGAAACCA 3294

S288C_SWH1 GAAACTTATTCCTGGAAAAAACCAAATAACACTGTTATCGGTATTTTAATGGGGAAACCA 3287

************************************************************

RM11-1a_SWH1 CAAGTAGACAACAGTGGGGACGTCAAAGTCACAAACCATACCACAGGCGACTATTGTATG 3354

S288C_SWH1 CAAGTAGACAACAGTGGGGACGTCAAAGTCACAAACCATACCACAGGCGACTATTGTATG 3347

************************************************************

RM11-1a_SWH1 CTGCATTACAAAGCCCATGGCTGGACCTCAGCCGGTGCATATGAAGTCAGAGGTGAAGTA 3414

S288C_SWH1 CTGCATTACAAAGCCCATGGCTGGACCTCAGCCGGTGCATATGAAGTCAGAGGTGAAGTA 3407

************************************************************

RM11-1a_SWH1 TTCAACAAGGACGGTAAAAAATTATGGGTTCTTGGTGGGCATTGGAATGATTCCATTTAC 3474

S288C_SWH1 TTCAACAAGGACGATAAAAAATTATGGGTTCTTGGTGGGCATTGGAATGATTCCATTTAC 3467

************* **********************************************

RM11-1a_SWH1 GGGAAAAAAGTAACTGCTAGAGGCGGAGAACTGACATTAGACAGAATAAAAACGGCAAAT 3534

S288C_SWH1 GGGAAAAAAGTAACTGCTAGAGGCGGAGAACTGACATTAGACAGAATAAAAACGGCAAAT 3527

************************************************************

RM11-1a_SWH1 TCTGCCACGGGAGGACCAAAACTAGATGGGTCTAAGTTTCTGATATGGAAAGCAAATGAA 3594

S288C_SWH1 TCTGCCACGGGAGGACCAAAACTAGATGGGTCTAAGTTTCTGATATGGAAAGCAAATGAA 3587

************************************************************

RM11-1a_SWH1 AGGCCTTCAGTGCCATTTAATTTAACGTTGTTTGCATTGACTTTGAATGCTTTGCCACCC 3654

S288C_SWH1 AGGCCTTCAGTGCCATTTAATTTAACGTCGTTTGCATTGACTTTGAATGCTTTGCCACCC 3647

**************************** *******************************

RM11-1a_SWH1 CACTTGGTACCATATTTAGCACCCACAGATAGTCGTTTAAGGCCCGATCAAAGGGCTATG 3714

S288C_SWH1 CACTTGATACCATATTTAGCACCCACAGATAGTCGTTTAAGGCCCGATCAAAGGGCTATG 3707

****** *****************************************************

RM11-1a_SWH1 GAAAATGGTGAATACGATAAAGCTGCCGCGGAAAAGCATCGTGTTGAAGTAAAACAAAGG 3774

S288C_SWH1 GAAAATGGTGAATACGATAAAGCTGCCGCGGAAAAGCATCGTGTTGAAGTAAAACAAAGG 3767

************************************************************

RM11-1a_SWH1 GCAGCAAAAAAAGAAAGGGAACAAAAAGGAGAAGAATACAGACCTAAGTGGTTTGTCCAG 3834

S288C_SWH1 GCAGCAAAAAAAGAAAGGGAACAAAAAGGAGAAGAATACAGACCTAAGTGGTTTGTCCAG 3827

************************************************************

RM11-1a_SWH1 GAGGAGCACCCCGTTACCAAAAGTCTATACTGGAAATTTAATGGAGAGTATTGGAACAAA 3894

S288C_SWH1 GAGGAGCACCCCGTTACCAAAAGTCTATACTGGAAATTTAATGGAGAGTATTGGAACAAA 3887

************************************************************

RM11-1a_SWH1 AGAAAAAATCATGACTTTAAAGATTGTGCTGATATTTTCTAA 3936

S288C_SWH1 AGAAAAAATCATGACTTTAAAGATTGTGCTGATATTTTCTAA 3929

******************************************
